# New insights on the biology of swine respiratory tract mycoplasmas from a comparative genome analysis

**DOI:** 10.1186/1471-2164-14-175

**Published:** 2013-03-14

**Authors:** Franciele Maboni Siqueira, Claudia Elizabeth Thompson, Veridiana Gomes Virginio, Taylor Gonchoroski, Luciano Reolon, Luiz Gonzaga Almeida, Marbella Maria da Fonsêca, Rangel de Souza, Francisco Prosdocimi, Irene Silveira Schrank, Henrique Bunselmeyer Ferreira, Ana Tereza Ribeiro de Vasconcelos, Arnaldo Zaha

**Affiliations:** 1 Centro de Biotecnologia, Universidade Federal do Rio Grande do Sul (UFRGS), Porto Alegre, Brazil; 2 Laboratório de Bioinformática. Laboratório Nacional de Computação Científica. Petrópolis, Rio de Janeiro, Brazil; 3 Programa de Pós-Graduação em Biologia Celular e Molecular. Centro de Biotecnologia UFRGS, Porto Alegre, Brazil; 4 Programa de Pós-Graduação em Ciências Biológicas - Bioquímica. UFRGS, Porto Alegre, Brazil; 5 Departamento de Biologia Molecular e Biotecnologia, Instituto de Biociências. UFRGS, Porto Alegre, Brazil; 6 Departamento de Bioquímica Médica. Centro de Ciências da Saúde, Universidade Federal do Rio de Janeiro, Rio de Janeiro, Brazil

**Keywords:** Mycoplasma, Comparative genomics, Adhesins, Swine respiratory tract

## Abstract

**Background:**

*Mycoplasma hyopneumoniae*, *Mycoplasma flocculare* and *Mycoplasma hyorhinis* live in swine respiratory tracts. *M. flocculare*, a commensal bacterium, is genetically closely related to *M. hyopneumoniae*, the causative agent of enzootic porcine pneumonia. *M. hyorhinis* is also pathogenic, causing polyserositis and arthritis. In this work, we present the genome sequences of *M. flocculare* and *M. hyopneumoniae* strain 7422, and we compare these genomes with the genomes of other *M. hyoponeumoniae* strain and to the a *M. hyorhinis* genome. These analyses were performed to identify possible characteristics that may help to explain the different behaviors of these species in swine respiratory tracts.

**Results:**

The overall genome organization of three species was analyzed, revealing that the ORF clusters (OCs) differ considerably and that inversions and rearrangements are common. Although *M. flocculare* and *M. hyopneumoniae* display a high degree of similarity with respect to the gene content, only some genomic regions display considerable synteny. Genes encoding proteins that may be involved in host-cell adhesion in *M. hyopneumoniae* and *M. flocculare* display differences in genomic structure and organization. Some genes encoding adhesins of the P97 family are absent in *M. flocculare* and some contain sequence differences or lack of domains that are considered to be important for adhesion to host cells. The phylogenetic relationship of the three species was confirmed by a phylogenomic approach. The set of genes involved in metabolism, especially in the uptake of precursors for nucleic acids synthesis and nucleotide metabolism, display some differences in copy number and the presence/absence in the three species.

**Conclusions:**

The comparative analyses of three mycoplasma species that inhabit the swine respiratory tract facilitated the identification of some characteristics that may be related to their different behaviors. *M. hyopneumoniae* and *M. flocculare* display many differences that may help to explain why one species is pathogenic and the other is considered to be commensal. However, it was not possible to identify specific virulence determinant factors that could explain the differences in the pathogenicity of the analyzed species. The *M. hyorhinis* genome contains differences in some components involved in metabolism and evasion of the host’s immune system that may contribute to its growth aggressiveness. Several horizontal gene transfer events were identified. The phylogenomic analysis places *M. hyopneumoniae, M. flocculare* and *M. hyorhinis* in the hyopneumoniae clade.

## Background

Mycoplasmas belong to the class Mollicutes, which is a taxon of bacteria that is characterized by the absence of a cell wall, a relatively small genome size and a strong dependence on nutrients supplied by the host environment [1]. More than 120 mycoplasma species have been described, and although they display diverse life styles, most of the species are parasitic, implying the occurrence of different mechanisms by which they interact with host cells. Several mycoplasmas associate with their host cells through adhesins, while others may also invade cells [2-8]. Among mycoplasmas, several species are responsible for human, animal and plant diseases, but some species are considered commensal organisms [1].

*Mycoplasma hyopneumoniae*, *Mycoplasma flocculare* and *Mycoplasma hyorhinis* are the most important species that have been identified in porcine respiratory systems [9-11]. Based on a 16S rRNA sequence comparison, *M. hyopneumoniae* and *M. flocculare* are known to be closely related [12]. *M. hyopneumoniae* is the etiological agent of porcine mycoplasmal pneumonia, while *M. hyorhinis*, which causes polyserositis and arthritis, is also frequently found in swine respiratory tracts [13]. *M. flocculare* is also widespread in swine herds, but no disease has been associated with this species [14]. *M. hyopneumoniae* can adhere to the cilia of tracheal epithelial cells and causes damage. Although *M. flocculare* can also adhere to cilia, no resulting damage has been observed, suggesting that *M. hyopneumoniae* and *M. flocculare* may possess different adhesins, facilitating the recognition of different receptor sites on the cilia [15]. Additionally, while *M. flocculare* is restricted to the swine respiratory tract, *M. hyopneumoniae* and *M. hyorhinis* can also colonize other sites, such as cardiac or joint tissues [14,16]. These bacteria can even colonize different hosts; *M. hyorhinis* has been detected in human carcinoma tissues [17,18]. The genetic maps of *M. flocculare* ATCC 27716 and *M. hyopneumoniae* strain J have been compared, revealing that at least three chromosomal inversions have occurred since the divergence of both species [19].

In recent years, the genomes of several mycoplasma species have been sequenced. The absence of several metabolic pathways, which was suggested by genetic and biochemical studies [1], has been confirmed at the genome sequence level. Among the swine-infecting mycoplasmas, the genomes of *M. hyopneumoniae* (four strains), *M. hyorhinis* (four strains) and *Mycoplasma suis* (two strains) have been sequenced, facilitating the comparison of metabolic pathways and evidencing specific mechanisms that can be utilized to survive in different host environments [20-28].

Because the genome sequences of *M. flocculare* and *M. hyopneumoniae* strain 7422 have now been completed, the current study presents a comprehensive comparison of the *M. flocculare*, *M. hyopneumoniae* and *M. hyorhinis* genomes. These three mycoplasma species can inhabit swine respiratory tracts. We have assessed the overall genome organizations including analyses of the open reading frame (ORF) clusters (OCs), inversions and rearrangements, and coding capacities, including analyses of encoded metabolic pathways and surface protein repertoires. Potential mechanisms of interaction with host cells are evidenced, and their implications on pathogenicity are discussed. Additionally, a phylogenomic approach using 32 mycoplasma genomes (including two that are reported here for the first time) was implemented to reconstruct the evolutionary history of the swine mycoplasma genomes, individual genes and/or portions of their genomes, including horizontal gene transfer analysis.

## Results and discussion

### General genome features of *M. flocculare* and *M. hyopneumoniae* 7422

The *M. flocculare* and *M. hyopneumoniae* 7422 genomes are composed of single, circular chromosomes of 763,948 and 899,887 bp with 28.9% and 28.4% GC contents, respectively. The *M. flocculare* genome, which by assembly remained with 13 gaps, contains 585 coding sequences (CDSs), of which 356 have known functions and 229 are annotated as hypothetical. The *M. hyopneumoniae* 7422 genome, completely closed in one contig by assembly, comprises 692 CDSs, of which 414 correspond to proteins with known functions and 278 are annotated as hypothetical. The protein-coding regions occupy approximately 87% of each *M. flocculare* and *M. hyopneumoniae* 7422 chromosome, and the average ORF length is 1,145 bp. Each genome contains one gene encoding the ribosomal RNAs (rRNAs) 16S and 23S, one gene encoding rRNA 5S and 30 genes encoding the transfer RNAs (tRNAs) representing all 20 amino acids. The general genome features of the five strains, *M. hyopneumoniae* (7422, 7448, J, 232 and 168), *M. flocculare*, and *M. hyorhinis* HUB-1, were compared in this study and are listed in Table 1.

**Table 1:** Comparison of general features of different mycoplasmas species and strains

Organism^*^							
	MHP 7422	MHP 7448	MHP J	MHP 232	MHP 168	MFL	MHR HUB-1
**Total length (bp)**	899,887	920,079	897,405	892,758	925,576	763,948	839,615
**G + C content (%)**	28.4	28.5	28.5	28.6	28.4	28.9	25.8
**Total no. CDSs**	692	716	690	692	695	585	654
**Average CDS length**							
**(bp)**	1,147	1,146	1,167	1,164	1,071	1,145	1,092
**Known proteins**	414	418	410	304	354	356	489
**Hypothetical proteins**	278	298	280	388	341	229	165
**No. of rRNAs**	3	3	3	3	3	3	3
**No. of tRNAs**	30	30	30	30	30	30	30

*Abreviations: MHP = *M. hyopneumoniae*; MFL = *M. flocculare*; MHR = *M. hyorhinis*

Among the *M. flocculare* and *M. hyopneumoniae* 7422 CDSs that encode proteins with known functions, 380 and 403 CDSs, respectively, were classified into COG families comprising 18 functional categories (Table 2). A functional classification based on the KEGG [29] analysis assigned 351 and 371 CDSs from *M. flocculare* and *M. hyopneumoniae* 7422, respectively, into 15 different categories (Table 2). The performance differences produced by COG with respect to KEGG may be attributable to the presence of paralogs. As expected, the general genomic features and similarities in all of the COG and KEGG categories were strikingly similar between *M. flocculare*, *M. hyopneumoniae*, and *M. hyorhinis*, which commonly exhibited small genome sizes, high AT contents, and no two signal transduction proteins (Table 2).

**Table 2:** **Comparison of *Mycoplasma *sp. genomes statistics using KEGG classification**

**Category/Organism**	**MHP 7422**		**MHR**		**MHP 7488**		**MHP J**		**MFL**	
	**Number**	**%**	**Number**	**%**	**Number**	**%**	**Number**	**%**	**Number**	**%**
Carbohydrate metabolism	83	22.4	73	17.3	82	22.2	82	22.4	73	20.8
Energy metabolism	20	5.4	22	5.2	19	5.1	20	5.5	20	5.7
Lipid metabolism	9	2.4	11	2.6	9	2.4	9	2.5	7	2
Nucleotide metabolism	47	12.7	44	10.4	46	12.5	45	12.3	46	13.1
Amino Acid metabolism	14	3.8	15	3.6	14	3.8	14	3.8	12	3.4
Metabolism of Other Amino Acids	7	1.9	8	1.9	7	1.9	7	1.9	7	2
Glycan Biosynthesis and Metabolism	1	0.3	5	1.2	2	0.5	1	0.3	2	0.6
Metabolism of Cofactors and Vitamins	9	2.4	14	3.3	9	2.4	8	2.2	8	2.3
Metabolism of Terpenoids and Polyketides	5	1.3	1	0.2	5	1.4	5	1.4	5	1.4
Membrane Transport	36	9.7	38	9	36	9.8	36	9.8	32	9
Folding. Sorting and Degradation	10	2.7	14	3.3	10	2.7	10	2.7	9	2.6
Replication and Repair	45	12.1	44	10.4	42	11.4	42	11.5	44	12.5
Transcription	3	0.8	3	0.7	3	0.8	3	0.8	3	0.9
Translation	72	19.4	98	23.2	73	19.8	72	19.7	73	20.8
Biosynthesis of Other Secondary Metabolites	8	2.2	2	0.5	9	2.4	9	2.5	7	2
Cell Motility	0	0	0	0	0	0	0	0	0	0
Signal Transduction	0	0	0	0	0	0	0	0	0	0
**TOTAL**	371		392		369		366		351										

Abbreviations as in Table 1.

**Figure 1:**
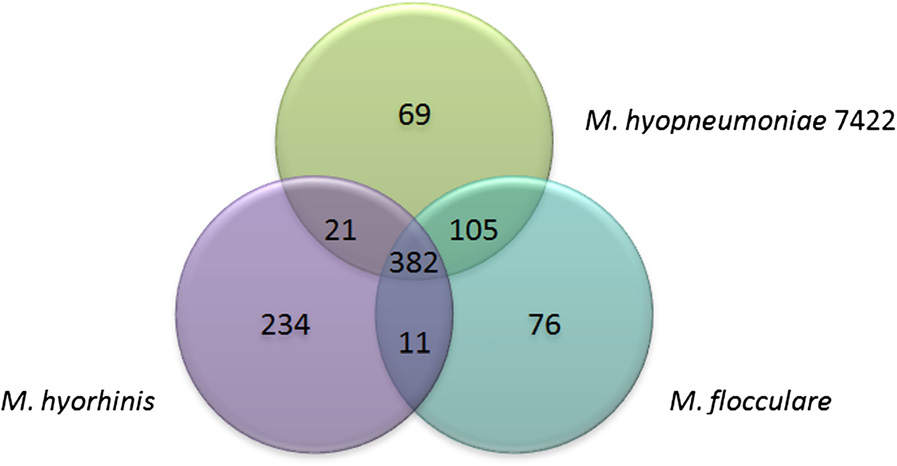
**Venn diagram showing the distribution of *M. hyopneumoniae *7422*, M. flocculare *and *M. hyorhinis *CDSs. **Venn diagram identifying the total number of common and exclusive CDSs for each genome.

To identify the genes that constitute the core and pan-genome of *M. flocculare*, *M. hyopneumoniae*, and *M. hyorhinis*, we took advantage of the bidirectional best hit (BBH) approach and plotted the data in a Venn diagram (Figure 1). We identified a considerable number of unique (i.e., organism-specific) genes in *M. hyorhinis* that may underline the phenotypic differences between this species and *M. flocculare* and *M. hyopneumoniae*. Including the repertoire of surface proteins (discussed later) and the inositol metabolism pathway, we identified 76 genes that are unique to *M. flocculare*, 69 to *M. hyopneumoniae* and 234 to *M. hyorhinis*.

When compared to other sequenced strains of *M. hyopneumoniae*, the genome of strain 7422 displays a highly similar gene composition and organization, with the exception of the localization of the integrative conjugative element (ICEH), which is positioned from 139,715 to 162,049 bp in the 7422 genome and from 518,376 to 540,705 bp in the 7448 genome. The similarity between *M. hyopneumoniae* gene repertories was 88% approximately. The small, but significative difference in the *M. hyopneumoniae* 7422 genome is the presence of an exclusive region of genes encoding transposases, hypothetical proteins and an ortholog of subtilisin-like serine protease (positioned from 497,277 to 510,210 bp). In comparison to 7422 genome, just one exclusive region was found in the 7448 genome, which is composed of genes encoding hypothetical proteins (positioned from 746,315 to 757,309 bp).

### Comparison of OC organization in *M. flocculare, M. hyopneumoniae* and *M. hyorhinis* genomes

The gene-by-gene genome organization of *M. flocculare*, *M. hyopneumoniae* 7448 and *M. hyorhinis* HUB-1 was analyzed, and the gene localization patterns were compared to detect ORFs with order conservation. The ORF cluster composition, organization and localization in the genomes were analyzed to determine the conservation level among the OC organization. Two groups of ORF clusters were created for each species, the OC group (Additional files 1 and 2) and the monocistronic gene (mC) group (Additional file 3). The general features of the OCs organization in the *M. flocculare, M. hyopneumoniae* and *M. hyorhinis* genomes are shown in Table 3 and Additional file 4.

**Table 3:** **Features of the *M. flocculare, M. hyopneumoniae *and *M. hyorhinis *OCs organization**

**Features**	MFL*	MHP*	MHR*
Total length (base pairs)	772.687	920.079	839.615
Total No. of OCs (CDSs total)	114 (582)	117 (657)	98 (654)
Total No. of monocistronic group	51	34	34
Exclusives OCs	10	24	36

* Abbreviations as in Table 1.

A comparison of the OCs arrangements revealed a similar number of OCs among the *M. flocculare*, *M. hyopneumoniae* and *M. hyorhinis* genomes (Table 3). This result suggests that gene organization in *M. flocculare* and *M. hyorhinis* also occur preferably in clusters as found in *M. hyopneumoniae*[30]. Moreover, as previously described for *M. hyopneumoniae*[30], the overall ORF distribution within the OCs in *M. flocculare* and *M. hyorhinis* is highly variable with respect to the number of ORFs and the functional categories of the encoded products (Additional files 1 and 2).

An analysis of the mC group revealed a different ORF number in the *M. flocculare* genome when compared to the organization in the *M. hyopneumoniae* and *M. hyorhinis* genomes. There were 51, 34, and 30 mCs in the *M. flocculare*, *M. hyopneumoniae* and *M. hyorhinis* genomes, respectively (Additional file 3). Among all of the mCs, seventeen mCs were shared only by the *M. flocculare* and *M. hyopneumoniae* genomes. However, only the CDSs encoding an O-sialoglycoprotein endopeptidase (*gcp*) and an excinuclease ABC subunit C (*uvrC*) were found to display monocistronic organization in the three genomes (Additional file 5).

A detailed analysis of the organization of each OC demonstrated a high level of conservation between the *M. flocculare* and *M. hyopneumoniae* genomes (Additional file 4). Approximately 78% and 46% of the OCs from *M. flocculare* display total or partial conserved gene distribution when compared to the OCs of *M. hyopneumoniae* and *M. hyorhinis*, respectively (Table 4; Additional file 4). Moreover, a comparative analysis of OC cluster organization among the three mycoplasma species revealed the presence of 12 OCs with complete similarity with respect to the ORF repertoires (synteny was not always detected). The majority of these OCs (seven OCs) were composed of two ORFs, with increasing numbers of up to five ORFs. These data are consistent with previous results that suggested that the majority of gene clusters in diverse organisms are formed by a string of two to four genes [30,31].

**Table 4:** **Comparison of OCs organization in *M. flocculare, M. hyopneumoniae *and *M. hyorhinis *genomes**

**ORF Clusters features**	MFL x MHP*	MFL x MHR*	MFL x MHP x MHR*
OCs 100% conserved	44	17	12
OCs Partially conserved	44	35	33
OCs Without conservation	29	62	-

* Abbreviations as in Table 1.

Another group of noteworthy OCs was the group classified as partially conserved among the three mycoplasma species. The number of partially conserved gene-order clusters in different genome pairs is shown in Table 4. The *M. flocculare* and *M. hyopneumoniae* genomes shared 44 OCs in which the gene string was partially conserved, and 33 OCs of the 44 OCs were classified as partially conserved in all three analyzed species. It is well known that only a few operons are conserved in most bacterial genomes; the classical example of conserved organization involves the ribosomal protein operons [32]. However, a detailed analysis of the 33 partially conserved OCs revealed gene context conservation in the ribosomal operons (see OC_7448_28 in Additional file 4) and in other clusters, such as clusters containing the chromosomal replication initiation protein (DnaA) (see OC_7448_01 in Additional file 4) and the OC containing the cell division protein MraZ (see OC_7448_67 in Additional file 4). In general, the similarity of the gene order (total or partial) among the prokaryotic genomes is maintained via the horizontal transfer of a chromosomal region. Our results suggest that individual genome pairs, such as *M. flocculare* and *M. hyopneumoniae* or *M. flocculare* and *M. hyorhinis*, share several OCs, which can partially be attributed to horizontal gene transfer.

A detailed, genomic-scale analysis of the OC organization in *M. flocculare* and *M. hyopneumoniae* demonstrated that species-specific differences are not present in genes with known function and/or related with pathogenicity (Additional file 6A-D). Apparently the 24 OCs exclusive to *M. hyopneumoniae* (not found in the *M. flocculare* genome) encode hypothetical proteins, transport-related proteins, myo-inositol utilization proteins, the integrative conjugative element (ICEH) and an additional copy of the P97 protein (Additional file 6A). In the *M. hyorhinis* genome, 35 OCs were unique to this species (Table 3; Additional file 6D), and the majority of the ORFs encode hypothetical proteins or products related to variable surface lipoproteins (*vlp* genes), which have been described as being involved in a complex system involving bacterial-host interactions [33].

### Rearrangements in the *M. flocculare, M. hyopneumoniae* and *M. hyorhinis* genomes

A detailed comparative analysis of genome organization is needed to understand the evolutionary dynamics of prokaryotic genomes. Therefore, a comparative genomic analysis was performed using the *M. flocculare* contigs (MFL contigs) and *M. hyopneumoniae* genome, considering the ORF string organization and OC distribution (Additional file 7A-B). Comparisons were also performed between MFL contigs and the *M. hyorhinis* genome (Additional file 7A-B); however, in this case, both the global alignment and gene-by-gene alignment were not applicable, possibly due to the large number of transpositions and inversions that have occurred in these genomes.

In the comparison between *M. flocculare* and *M. hyopneumoniae*, 22 regions (with lengths ranging from 2 to 75 kb) were identified as being involved in inversions or rearrangements (Additional file 7B). Among these regions, only eight showed major rearrangements, although the OC organization was maintained. Notably, OCs containing several of the genes encoding pathogenicity-related proteins, such as lipoproteins and adhesins, were located within these regions. For instance, major rearrangements were observed in gene clusters encoding P97, P102 (MFL contig 13), P60, P69, P37 (MFL contig 34), P216, P76 (MFL contig 20) and the 46 K surface antigen precursor (MFL contig 4). Genes encoding transposases were found adjacent to some of the inverted segments, such as in MFL contig 23 (containing the P146 and MgPa proteins) and MFL contig 15 (containing the P97-like and P102-like adhesins), suggesting a possible role of these transposases in the rearrangements. Our findings in the comparison between the *M. flocculare* and *M. hyopneumoniae* genomes are similar to the situation found in the genomes of the two closely related species *Mycoplasma pneumoniae* and *Mycoplasma genitalium*, whose genomes can be divided into segments with highly conserved gene organization, although the segments are arranged differently [34].

The lack of gene-order conservation beyond the operon level even between relatively closely related species has been previously described [32]. Apparently, in closely related mycoplasmas, such as *M. flocculare* and *M. hyopneumoniae*, large-scale gene-order conservation is observed, although genome collinearity is disrupted at some points. Chromosomal rearrangements are generally caused by homologous recombination between repeated sequences within the genome [19,35]. Although the number of genes involved in DNA repair and recombination in mycoplasmas is relatively small [36], the gene encoding RecA was found in all sequenced mollicute genomes. Recently, the importance of RecA in the antigenic and phase variation of the MgpB and MgpC adhesins in *M. genitalium* has been demonstrated [37].

### Repertoire of surface proteins encoded by *M. flocculare, M. hyopneumoniae* and *M. hyorhinis*

A comparative *in silico* survey of the repertoire of encoded surface proteins was performed between the genome of the non-pathogenic *M. flocculare* and the genomes of two pathogenic mycoplasma species that are found in this tissue in the swine respiratory tract, *M. hyopneumoniae* (represented by the 7448 strain) and *M. hyorhinis* (represented by the HUB-1 strain). The results of this survey are summarized in Additional files 8 and 9, and the complete generated datasets are presented in Additional files 10, 11 and 12. Of the total of 585 *M. flocculare* CDSs, 277 (47.5%) were predicted to encode surface proteins; this number was similar to that of *M. hyopneumoniae* 7448 (292 out of 716; 44.4%) and higher than that of *M. hyorhinis* HUB-1 (247 out of 654, 37.7%)

(Additional file 10A-C). The proportion of CDSs encoding surface proteins in these species is considerably large considering their small genome sizes. From these surface protein sets, 28 (10.1%), 42 (14.4%), and 44 (17.8%) CDSs are unique to *M. flocculare*, *M. hyopneumoniae* and *M. hyorhinis*, respectively, with respect to the other two species (Additional file 8; Additional file 11A-C).

The repertoire of *M. flocculare* surface proteins that is not shared with the other two species consists exclusively of hypothetical proteins; although those of *M. hyopneumoniae* and *M. hyorhinis* are predominantly composed of hypothetical proteins (73.8% and 59%, respectively), they also include some proteins with assigned functions. Among these proteins with predicted functions, *M. hyopneumoniae* includes proteins involved in myo-inositol catabolism, a permease and a protein encoded by the integrative conjugative element (ICEH), and *M. hyorhinis* includes some variable antigens, secretory system components, transporters and lipoproteins.

**Figure 2:**
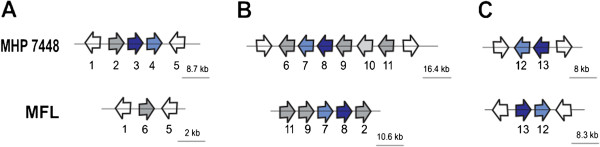
**P97 and P102 gene organization contexts in the *M. flocculare *and *M. hyopneumoniae *7448 genomes. **(**A**) P97 copy 1 ORF cluster organization. (**B**) P97 copy 2 ORF cluster organization. **(C) **P97-like ORF cluster organization. The arrows represent the ORFs (not to scale) and indicate the transcriptional direction. The dark-blue arrows represent the P97 ORFs, and the light-blue arrows represent the P102 ORFs. The white arrows represent the ORFs that are at the limits of the OC. The numbers from one through thirteen represent the ORF name and the names of its orthologous as follows: 1- *rpsJ*; 2- MF1418 and MHP0197; 3- P97 copy 1; 4- P102 copy 1; 5- MF0249 and MHP0200; 6- MF0247 and MHP0106; 7- P102 copy 2; 8- P97 copy 2; 9- *gyrB*; 10- transposase; 11- *pfkA*; 12- P102-like; and 13- P97-like.

Based on an E-value cutoff threshold of 1e-6 to define orthology, nearly 90% of the repertoire of *M. flocculare* surface proteins is shared with *M. hyopneumoniae* and/or *M. hyorhinis* (Additional file 8; Additional file 12). Searches using more stringent conditions resulted in not more than a 20% reduction in the numbers of identified orthologs (data not shown). These results are indicative of physiological similarities that would be consistent with the adaptation to the same host environment. Of the shared proteins, approximately 40% have unknown functions (hypothetical proteins), while the other 60% consist of proteins with assigned functions in at least one of the compared species. Notably, many of these shared proteins (46 proteins, marked in bold in Additional file 12) correspond to putative pathogenicity-related genes in *M. hyopneumoniae* and/or *M. hyorhinis*; these proteins include several lipoproteins and adhesins that are thought to play a role in virulence despite of the non-pathogenic nature of *M. flocculare* and the pathogenicity differences between *M. hyopneumoniae* and *M. hyorhinis*. For instance, *M. flocculare* contains orthologs for the P97 copy 2 and for the P97-like adhesins of *M. hyopneumoniae*, although it lacks an ortholog for P97 copy 1. The genomic organization of the P97 copy 2 and P97-like ortholog CDSs are similar in both species with respect to gene clustering (Figure 2); this result suggests that P97 copy 2 and P97-like are ancestral P97 paralogs and that they were present in a common ancestor to *M. flocculare* and *M. hyopneumoniae*. A second duplication event, which originated the *M. hyopneumoniae* P97 copy 1, would have occurred after the divergence of *M. hyopneumoniae* from *M. flocculare*.

Additional file 13 lists some adhesins that have been associated with pathogenicity and have been experimentally analyzed [38-43] in *M. hyopneumoniae* 232. The *M. hyopneumoniae* 7448 and *M. flocculare* genomes contain orthologs for all the adhesins with the aforementioned exception of one copy of the P97 and P102 proteins that are absent in *M. flocculare* (also shown in Figure 2). The gene organization and location was analyzed and, as described in Additional file 13, the regions containing these orthologs are involved in inversions or rearrangements (Additional file 7B) in both mycoplasma species. Specifically, three important adhesins (P216, P159 and P60) display highly conserved gene organization between *M. hyopneumoniae* 232, 7422, 7448 and 168 strains, but they display inversions and rearrangements in *M. flocculare.* The participation of *M. hyopneumoniae* adhesins in host-cell adhesion is a complex process involving specific cleavage events [44]. The set of *M. flocculare* genes that may be involved in adhesion may not be complete, which would explain the differences in host-cell adhesion with respect to *M. hyopneumoniae*[18]. These results may explain the presence of orthologs in *M. flocculare* despite its lack of pathogenic capacity.

The presence of surface virulence determinants even in the non-pathogenic *M. flocculare* and in a non-pathogenic strain of *M. hyopneumoniae* (J strain) [21,45] suggests that their roles in pathogenicity may depend on their expression levels and/or post-translational processing, which may vary [46]. Differences in virulence between species and strains can also be associated with the presence of variants of these proteins with or without some functional domains that are associated with features such as adhesion capacity or antigenicity, which has previously been described for several *M. hyopneumoniae* and *M. hyorhinis* virulence factors [40,41,47,48]. For instance, the *M. flocculare* P97 copy 2 and P97-like orthologs present relatively high overall identities to their *M. hyopneumoniae* counterparts (53% and 57%, respectively), but in the case of P97 copy 2, the *M. flocculare* ortholog lacks a domain (R1) regarded to be important for virulence in *M. hyopneumoniae*; instead, it contains a second R2 domain (Additional file 14). These R1 and R2 repeats are absent from the *M. flocculare* P97-like protein and its orthologs from *M. hyopneumoniae* (P97-like adhesin) and *M. hyorhinis* (P95).

Cell-surface features with implications for virulence may also reside in the 10 to 18% of the repertoires of surface proteins that are not shared between *M. flocculare*, *M. hyopneumoniae* and *M. hyorhinis*. However, because all (in the case of *M. flocculare*) or most (in the cases of *M. hyopneumoniae* and *M. hyorhinis*) of these unshared CDS products are hypothetical or conserved hypothetical proteins, their potential contributions to pathogenicity remain elusive. However, considering the nature of the unshared CDS products annotated in *M. hyopneumoniae* and *M. hyorhinis*, these ‘exclusive’ and unknown proteins are likely to include players of processes that are important for pathogen-host interactions, such as proteins involved in secretion, the uptake of certain molecules, conjugation and immune evasion/modulation. The variation in *M. hyorhinis* surface lipoproteins (Vlp) is considered important to protect the organism from the humoral response and may be a primary adaptive strategy for immune evasion during infection and disease [49,50]. Therefore, at least some of these proteins are expected to compose a portion of the repertoire of determinants of virulence or avirulence for each species or strain.

An additional comparison of the repertoires of surface proteins from *M. flocculare*, *M. hyopneumoniae* and *M. hyorhinis* was performed based on the COG classification of the predicted surface protein sets for each species (see Additional file 10). The produced COG functional profiles of surface proteins for the three species are summarized in Additional file 9. According to the COG, the functional surface protein profile for *M. flocculare* is similar to those for *M. hyopneumoniae* and *M. hyorhinis*; similar numbers of proteins were assigned to each category for the three species. This similarity was observed even for the U and M categories, which include secretion system components (whose repertoires are virtually equivalent for the three species; data not shown), and for the no-COG category, which included 45-53% of the proteins, most of which (82.2% for *M. flocculare*, 86.6% for *M. hyopneumoniae*, and 64.8% for *M. hyorhinis*) were represented by hypothetical proteins or in the additional category of antigen, adhesin or lipoprotein, in which proteins were included based on their prior immunological or functional characterization according to published studies. Overall, the surface protein set for *M. flocculare*, taken from the correspondent COG profile, was shown to be very similar to those of *M. hyopneumoniae* and *M. hyorhinis*. This result suggests that the species have equivalent genetic backgrounds for metabolic and growth processes. Such functional similarities may be the result of common selective pressures associated with the colonization of the same environment (i.e., the swine respiratory tract).

The L (replication, recombination and repair) and V (defense mechanisms) categories displayed differences; *M. flocculare* (and *M. hyorhinis*) contained approximately half of the number of proteins as *M. hyopneumoniae*. Protein sets assigned to the L category are heterogeneous, and the relative excess of proteins in *M. hyopneumoniae* corresponds to transposases that cannot be found in either *M. flocculare* or *M. hyorhinis*. Conversely, category V is enriched with ATP-binding cassette (ABC) transporter system proteins related to defense mechanisms, such as the *M. hyorhinis* ABC-type multidrug-like transport system ATP-binding proteins and their orthologs in *M. flocculare* and *M. hyopneumoniae*. The remaining transporters, including ABC and non-ABC transporter system components (such as those from the phosphotransferase system; PTS) appear in other COG categories, such as E, G, R or P. However, the overall number of transporters unrelated to defense mechanisms (non-V) is roughly equivalent in the three species, with 50, 49, and 47, in the *M. flocculare*, *M. hyopneumoniae* and *M. hyorhinis* surface protein sets, respectively. Although *M. hyorhinis* have 19 genes encoding transposases, and *M. hyopneumoniae* 9 genes, notably, the presence of transposases among *M. hyopneumoniae* predicted surface proteins may be an artifact due to the occurrence of helical structures in these enzymes [51]; these helices can be misidentified as transmembrane domains. However, the differential presence of at least some transporter system components is indicative of certain *M. hyopneumoniae* capabilities that are unavailable in both *M. flocculare* and *M. hyorhinis*. A larger number of transport proteins is usually related to a species’ capacity to persist in different tissue environments [52], but this phenomenon does not seem to apply to *M. flocculare*, *M. hyopneumoniae* or *M. hyorhinis* because they share a large portion of the transporter repertoire. This situation is similar to that observed for secretory system components. However, according to the COG (see below), *M. hyopneumoniae* has approximately two-fold more transporters associated with defense mechanisms than the other two species. In this aspect, *M. hyopneumoniae* is more similar to *Mycoplasma bovis*[53], which infects the respiratory tract and breast and joint tissues of bovines, than to *M. flocculare* or *M. hyorhinis*. The implications of the larger *M. hyopneumoniae* repertoire of defense mechanisms proteins (COG V) for its survival in the swine respiratory tract have not yet been investigated.

### Phylogenomics and the phylogenetics of *Mycoplasmatacae*

**Figure 3:**
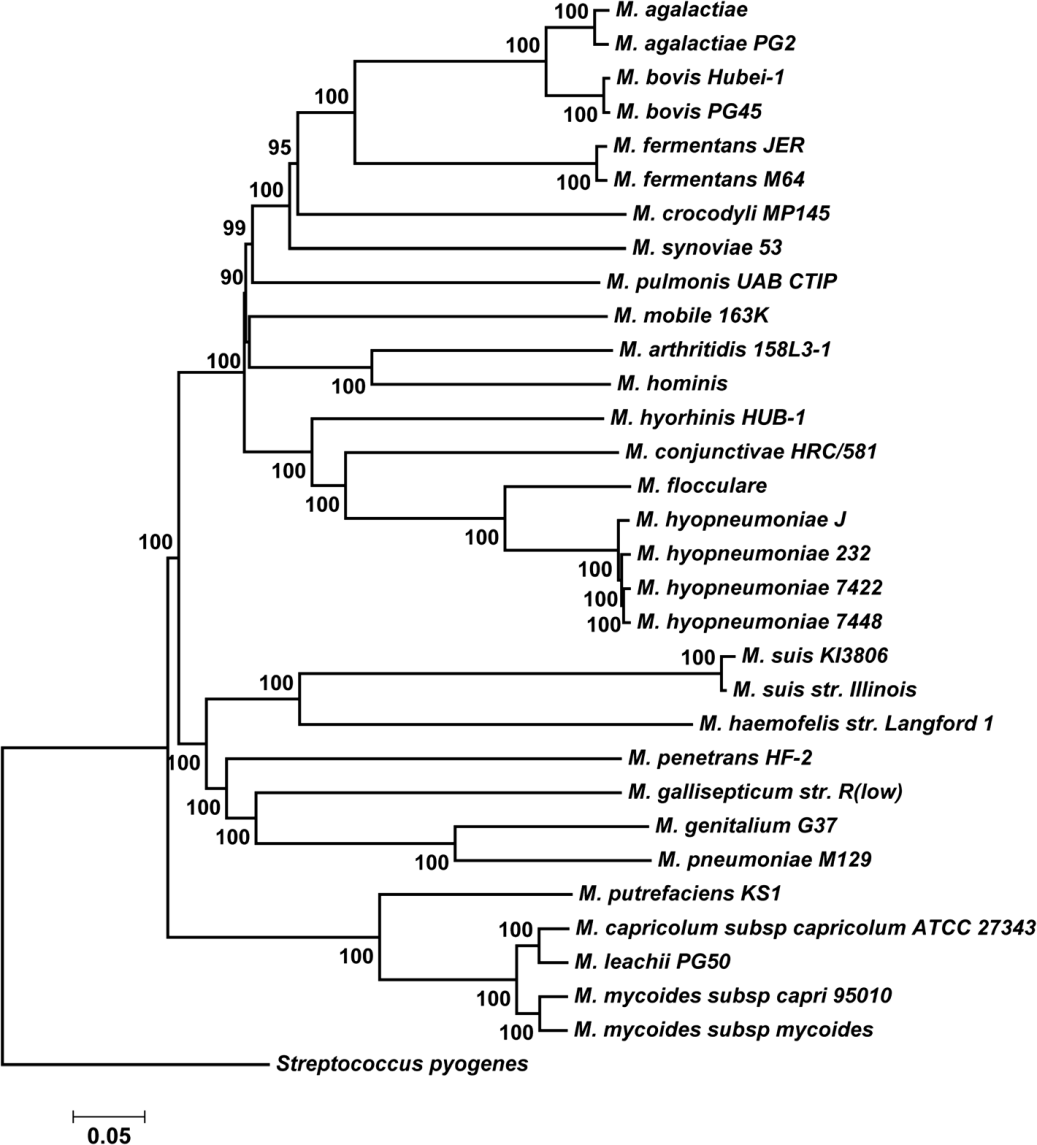
**Evolutionary history of mycoplasmas obtained through a phylogenomic approach.** The Neighbor-Joining method using the p-distance to compute the evolutionary distances and the pairwise deletion of gaps was implemented in the MEGA 5 software program. The percentage of replicate trees in which the associated taxa clustered together in the bootstrap test (1,000 replicates) is shown next to each branch. *Streptococcus pyogenes *was used as the outgroup.

From the entire set of 585 annotated *M. flocculare* genes, 179 gene sets were retrieved that contained at least one gene representative for each swine mycoplasma analyzed here (BLAST cut off E-10). Overall, 179 ortholog-like files representing different CDSs were concatenated, leading to an aligned file containing 104,097 amino acid residues. The neighbor-joining method (NJ) and maximum parsimony (MP) tree topologies did not differ significantly, especially when major clades were considered (Additional files 15, 16, 17; 18). There was consensus in several aspects (Figure 3). As expected, all of the *M. hyopneumoniae* strains formed a monophyletic clade. Additionally, the *M. hyopneumoniae* monophyletic clade was closely related to *M. flocculare*, with high bootstrap support. Finally, *M. hyorhinis* is basal to *Mycoplasma conjunctivae*, *M. flocculare* and *M. hyopneumoniae* in all of the phylogenomic trees.

The *Mycoplasmataceae* species were subdivided into the following clades: bovis (including *Mycoplasma agalactiae*, *M. bovis* and *Mycoplasma fermentans*), hominis (*Mycoplasma hominis* and *Mycoplasma arthritidis*), hyopneumoniae (*M. hyorhinis*, *M. conjunctivae*, *M. flocculare*, and *M. hyopneumoniae*), hemotrophic mycoplasma (*Mycoplasma suis* and *Mycoplasma haemofelis*), genitalium-pneumoniae (*Mycoplasma gallisepticum*, *Mycoplasma genitalium*, and *Mycoplasma pneumoniae*), and mycoides (*Mycoplasma putrefaciens*, *Mycoplasma capricolum*, *Mycoplasma leachii*, and *Mycoplasma mycoides*). All of these clades displayed high bootstrap values. The synoviae-pulmonis (*M. crocodyli*, *M. synoviae*, and *M. pulmonis*) group did not form a monophyletic cluster, but they are closely related to the bovis clade. *M. penetrans* HF-2 is near the genitalium-pneumoniae clade.

Our mycoplasma phylogenomic tree (Figure 3) corroborated the results that were obtained using the RNA polymerase beta subunit (rpoB), 16S-23S rRNA intergenic transcribed spacer region (ITS), and 16S rRNA genes [54,55]. *M. flocculare*, *M. hyopneumoniae* 7448, and *M. hyorhinis* HUB-1 were located in the hyopneumoniae clade.

When comparing the *M. flocculare*, *M. hyorhinis* HUB-1 and *M. hyopneumoniae* 7448 genomes, several paralog clusters were identified through the bidirectional best hit (BBH) approach, wherein a paralog cluster was defined as a gene set in which every gene is a BBH with at least one other element. Fourteen of these paralogs (DNA methylase, ATP synthase, ribulose-phosphate 3-epimerase, oligoendopeptidase F, single-strand binding protein, fructose-bisphosphate aldolase, dihydrolipoamide dehydrogenase, glucose-6-phosphate isomerase, lipoate-protein ligase, acyl-carrier-protein phosphodiesterase, lactate dehydrogenase, membrane nuclease lipoprotein, TrsE-like protein, and P97) were submitted to phylogenetic analyses to understand the evolutionary history of those paralogs.

Phylogenetic analyses of mycoplasma DNA methylases, which are enzymes that catalyze the transfer of a methyl group to DNA [56], contained ancient gene duplications in the hyopneumoniae group, leading each DNA methylase paralog to form a monophyletic group that included *M. flocculare* and *M. hyopneumoniae* (Additional file 19). Methylation, in addition to involvement in restriction systems, plays an important role in controlling gene expression, and it is one of the most significant DNA modifications [57]. The N^6^-adenine methylation is involved in bacterial gene regulation and virulence [58-60]. CpG motifs in bacterial DNA may play a significant pathogenic role in inflammatory lung disease because the proinflammatory effects can be reduced by DNA methylation [61]. Microarray analyses and RT-PCR have demonstrated that the deletion of a C^5^-cytosine methyltransferase in *Helicobacter pylori* strains can affect the expression of several genes related to motility, adhesion and virulence [57].

The ATP synthase phylogeny indicated that *M. flocculare*, *M. hyorhinis* HUB-1, and *M. hyopneumoniae* 7448 cluster according to the ATP synthase subunit, with the alpha subunit presenting a more complex evolutionary pattern (Additional file 20). This enzyme is required to synthesize adenosine triphosphate (ATP), providing energy to the cell. The paralogs found in mycoplasmas are related to different subunits that are required for enzymatic function [62].

Ribulose-phosphate 3-epimerase interconverts the stereoisomers ribulose-5-phosphate and xylulose-5-phosphate [63]. Its phylogeny revealed that ancient duplications occurred in the hyopneumoniae group. Other gene duplications responsible for the *M. hyorhinis* GDL-1 and *M. hyorhinis* HUB-1 split can be observed at the base of this *M. hyorhinis* clade (Additional file 21). Similarly, recent gene duplications can be observed in the *M. hyorhinis* clade in the oligoendopeptidase F (Additional file 22) and single-strand binding protein (Additional file 23) phylogenetic trees, which both contain high bootstrap support.

*M. flocculare* and *M. hyopneumoniae* 7448 contain two copies of fructose-bisphosphate aldolase, while *M. hyorhinis* HUB-1 contains only one copy. These duplications likely occurred prior to the diversification of the hyopneumoniae group (Additional file 24). The same process occurred during dihydrolipoamide dehydrogenase evolution, as shown in Additional file 25. *M. hyorhinis* HUB-1 contains two copies of the dimeric glycolytic enzyme glucose-6-phosphate isomerase, which catalyzes the reversible isomerization of glucose-6-phosphate and fructose-6-phosphate [64]. The lack of statistical confidence in some tree branches did not facilitate inferences regarding the evolutionary history of these copies in *M. hyorhinis* HUB-1 (Additional file 26).

Two lipoate-protein ligases are found in *M. hyopneumoniae* 7448 and *M. hyorhinis* HUB-1. They are more closely related to the enzymes from other microorganisms than to each other (Additional file 27). In *M. flocculare*, only one copy was identified. The same pattern was observed in the phylogenetic tree of acyl-carrier-protein phosphodiesterase (Additional file 28), which belongs to the hydrolase family and acts on phosphoric diester bonds [65]. The topology of the lactate dehydrogenase tree showed that the two *M. hyorhinis* HUB-1 protein copies are significantly different, which led the sequences to be grouped in distant clades (Additional file 29). A recent duplication of membrane nuclease lipoprotein resulted in a *M. hyorhinis* cluster containing the two *M. hyorhinis* HUB-1 copies. A unique copy of this specific protein was found in *M. flocculare* and *M. hyopneumoniae* 7448 (Additional file 30).

Two copies of the TrsE -like protein were identified in *M. hyopneumoniae* 7448, and one copy was identified in the *M. flocculare* and *M. hyorhinis* HUB-1 genomes. Each copy is more similar to other sequences in the *M. hyopneumoniae* strain than to one another (Additional file 31). Finally, P97 is an adhesin thought to play a role in virulence. Several copies have been detected in the hyopneumoniae group. The phylogenetic tree indicated that the copies of *M. flocculare* and *M. hyopneumoniae* 7448 are more closely related to the sequences from other species and strains than to each other (Additional file 32).

Although mycoplasmas contain reduced genomes, some paralogs are maintained in their genomes. A phylogenetic analysis was conducted to better understand the evolution of those paralogs. Gene copies are known to be preserved in a genome if the organism demands high levels of particular gene products. In other cases, positive selection can result in the diversification of the gene’s function, a process called neofunctionalization [66]. Additionally, subfunctionalization can lead to the loss of function, resulting in duplicated genes whose functions differ to some degree [67]. Even highly conserved genes may have slightly or very different functions, such as glycolytic enzymes such as fructose-bisphosphate aldolase (FBA), which have been described as complex, multifunctional proteins that perform non-glycolytic functions [68].

Some mycoplasma paralog proteins, such as lipoate- protein ligase may possess different functions or differ in substrate specificity. Otherwise, essential enzymes, such as ATP synthase, may maintain multiple gene copies because they encode different subunits that are required for enzymatic function, despite the recent finds of losses of this enzyme family in the common ancestor of Mollicutes [69]. According to the standard model of phylogenomics, there is a higher similarity among orthologs than paralogs [67]. The paralog genes initially display identical sequences and functions. However, the action of selective pressures and mutations lead to divergence in regulatory and coding sequences [70].

### Horizontal gene transfer

The species tree that was generated by the phylogenomic analysis was compared to the individual gene trees to investigate the occurrence of horizontal gene transfer (HGT) events. HGTs are an important source of genome innovation and evolution in prokaryotes [71], and it apparently also impacts Mycoplasmataceae evolution.

In mycoplasmas, we observed several HGT events (Additional file 33); some of the events occurred between *M. hyorhinis* HUB-1 and *M. conjunctivae* HRC. The events involving species belonging to the hyopneumoniae group occurred in ribosomal proteins, GTP-binding proteins, heat-shock proteins, DNA primase, signal recognition particle protein, ABC transporter ATP-binding proteins, phosphoesterases, cell division protein, elongation factor, fructose-biphosphate aldolase, DNA polymerase, glutamyl-tRNA synthetase, helicases, and hypothetical proteins. Regions encoding ABC transporters were likely transferred between *M. synoviae* and *M. gallisepticum* (Additional file 33), corroborating previously published results [21].

It is well-known that prokaryotes exchange genes in a sophisticated manner via lateral transfer, and bacterial phylogenies may also be viewed as a complex network of genomic exchange. However, the sequence-based methods implemented in the phylogenomic studies have yielded phylogenetic trees that are similar to rRNA trees, which were demonstrated in the current study. Consequently, lateral transfer events do not prevent the recovery of phylogenetic signals in prokaryotes, although they do add an extra source of noise [72].

### Metabolism overview

Mycoplasmas contain a reduced genome; therefore, they lack many metabolic pathways, particularly biosynthetic pathways, such as those involved in cell-wall production, de novo purine biosynthesis and the biosynthesis of amino acids [73]. These organisms also lack a functional tricarboxylic acid (TCA) cycle because they are extremely fastidious in their nutritional requirements and dependent on nutrients supplied by their hosts. They produce high levels of enzymes responsible for the degradation of nucleic acids and proteins and transporters to obtain the precursors of these macromolecules. Most mycoplasma species depend on the glycolytic pathway to generate ATP. Some species may produce ATP based on the reaction involving acetyl phosphate and ADP by acetate kinase, coupled with acetyl phosphate formation from acetyl-CoA by phosphate acetyl transferase. Acetyl CoA is formed by the pyruvate dehydrogenase complex [1,74]. Some mycoplasma species such as *M. hominis* and *M. arthritidis* produce ATP through arginine degradation, using the arginine dihydrolase pathway [75]. This pathway is absent in the mycoplasma genomes analyzed in this work. All of the genes that encode enzymes of the glycolytic pathway exist in the three species; however, some differences in gene copy number were observed. Two copies of the genes encoding fructose-bisphosphate aldolase exist in *M. hyopneumoniae* and *M. flocculare*, two copies of the gene encoding d-ribulose-5-phosphate 3 epimerase are present in *M. hyopneumoniae*, and two copies of the gene encoding glucose-6-phosphate isomerase are present in *M. hyorhinis*. The possible influence of the gene copy number on the physiology of the species is unknown. However, some glycolytic enzymes have been described as virulence factors, exhibiting functions unrelated to glycolysis, such as adhesion to the host cells, may contribute to the pathogenesis of mycoplasmas infections [7,68,76-79]. The evolutionary aspects of these paralogs are discussed in the “Phylogenomics and phylogenetics of *Mycoplasmatacae*” section.

It has been shown that nuclease activities can be detected in Mollicutes and that these activities are primarily associated with the membrane and may be essential for growth and survival [80]. Genes encoding nucleases or putative membrane-associated nucleases were found in the three analyzed *Mycoplasma* species. Two gene sets encoding membrane nucleases were observed in the three species. One set is represented by two copies of *mnu*A (MHR_0206 and MHR_0549) in the *M. hyorhinis* HUB-1 genome and a single copy in *M. hyopneumoniae* 7448 (MHP7448_0580), *M. hyopneumoniae* 7422 (MX03145) and *M. flocculare* (MF00420). Another set represented by two copies was observed in the genomes of the three species. The cell surface-exposed exonuclease (mhp379) from *M. hyopneumoniae* 232, a representative of the latter set, has been analyzed, and it has been proposed that the exonuclease activity of mhp379 may be important for importing nucleic acid precursors [81]. The presence of an extra copy of a nuclease gene in *M. hyorhinis* may represent a potential advantage of this species in acquiring nucleic acids precursors. Mycoplasmas cannot de novo synthesize purines and pyrimidines; therefore, they depend on salvage and interconversions to supply the cell with the nucleic acid precursors [82]. The three species contain a similar set of genes involved in purine (MHP7448_0084 - *M. hyopneumoniae*, MHR_0566 - *M. hyorhinis* and MF01198 - *M. flocculare*) and pyrimidine (MHP7448_0578 - *M. hyopneumoniae*, MHR_0640 - *M. hyorhinis* and MF00424 - *M. flocculare*) metabolism (See Additional file 4). However, *M. hyorhinis* contains genes encoding thymidylate synthetase (TS), allowing the conversion of dUMP to dTMP, and dihydrofolate reductase (DHFR), which catalyzes the reduction of dihydrofolate (DHF) to tetrahydrofolate (THF). The presence of TS and DHFR in *M. hyorhinis* may also contribute to its ability to overgrow the other *Mycoplasma* species in the swine respiratory tract [14].

## Conclusions

The comparative analyses of three mycoplasma species that inhabit the swine respiratory tract facilitated the identification of some characteristics that may promote the understanding of their different behaviors. The *M. hyopneumoniae* strain 7422 genome displays a similar organization as to the other previously described strains, but it contains rearrangements and an altered position of ICEH in the genome. The genomes of *M. hyopneumoniae* and *M. flocculare*, two closely related species, contain some blocks of synteny, but they also display many differences that may help to explain why one species is pathogenic and the other is commensal. However, it was not possible to correlate specific virulence determinant factors to the pathogenicity differences of the analyzed species. A large proportion of the repertoire of *M. flocculare* surface proteins is shared with *M. hyopneumoniae* and/or *M. hyorhinis,* which would be expected because the organisms may occupy the same niche. However, certain members of the p97 family are absent in *M. flocculare*, and some display sequence differences or lack domains that are considered to be important for host-cell adhesion. *M. hyorhinis* contains some metabolic genes that are absent in the other species, suggesting a possible advantage in the growth of this species. The differences in some components involved in evasion of the host immune system may also contribute to the aggressive growth of *M. hyorhinis*. The phylogenomic analysis confirmed previous results, placing *M. hyopneumoniae, M. flocculare* and *M. hyorhinis* in the hyopneumoniae clade. Several horizontal gene transfer events were identified, and several of them occurred between *M. hyorhinis* and *M. conjunctivae*.

## Methods

### Bacterial strains, culture conditions, and DNA isolation

*M*. *hyopneumoniae* strain 7422 was isolated from an infected swine in Lindóia do Sul, Santa Catarina, Brazil. *M. flocculare* (ATCC 27716) was acquired by Embrapa Suínos e Aves (Concórdia, Brazil) from the American Type Culture Collection. Both of the strains were cultivated in 5 mL of Friis medium [83] at 37°C for 48 h, and genomic DNA was extracted according to a standard protocol [84].

### Genome sequencing, assembly and annotation

For each species, one 454 shotgun library was prepared with approximately 5 μg of gDNA. The library construction, titration, emulsion PCR and sequencing steps were performed explicitly according to the manufacturer’s protocol. Sequencing was performed using Roche 454 GS FLX Titanium platform. *M. flocculare* was sequenced in one region of a two-region PicoTiterPlate (PTP), and *M. hyopneumoniae* 7422 was sequenced in two regions of an eight-region PTP. The contigs were assembled using the Newbler software program version 2.6 with the default parameters. The PCR assisted contig extension (PACE) strategy [85] was used for physical gap closure by the ends regions of gaps. For *M. hyopneumoniae* 7422 and *M. flocculare*, the estimated genome coverage for both genomes was 23X. The *M hyopneumoniae* 7422 genome was completely closed in one contig, and the *M. flocculare* genome retained 13 gaps.

The annotation and analysis of the sequences of both genomes were performed using the System for Automated Bacterial Integrated Annotation (SABIA) [86]. The comparative analysis was based on the Bidirectional Best Hits (BBH) [87] approach using the BLASTP (Basic Local Alignment Search Tool) [88] program to identify corresponding gene pairs recognized as the best hits in other genomes. All of the BLASTP searches were performed using the following parameters: an e-value of 10^-5^, query coverage of 60%, and positive similarity value of 50%. The comparative databank is available at http://www.genesul.lncc.br/comparative/.

To store and analyze the data, a databank was developed using the MySQL and Perl programming languages. This databank integrates tools and information from numerous biological databases, such as The Integrated Resource of Protein Domains and Functional Sites (Interpro) [89], Protein Subcellular Localization Prediction Tool (Psort) [90], Kyoto Encyclopedia of Genes and Genomes (KEGG) [29], Clusters of Orthologous Groups of Proteins (COG) [91], Transporter Classification Database (TCDB) [92], BLASTP of KEGG and UniProt/Swiss-Prot [93], facilitating several analyses, such as cluster with minimal genomes and clusters exclusives genes for each genome analyzed. In addition, the databank allows automatic genomic comparisons by bidirectional best hits (BBH) between five species. This whole-genome shotgun project has been deposited at DDBJ/EMBL/GenBank under the accession PRJNA65295 ID: 65295 for *M. flocculare* and PRJNA47327 ID: 47327 for *M. hyopneumoniae* 7422.

### *In silico* analysis of ORF clusters (OCs)

The prediction of OCs was performed by the Artemis Release 10.5.2 software program [94] according to previously established criteria [30]. The manual examination of the possible OCs in the *M. flocculare, M. hyorhinis* and *M. hyopneumoniae* genomes was established based on the occurrence of clusters with two or more tandem genes in the same DNA strand. This procedure was performed by a systematic annotation comparison of the protein sequences encoded in all of the ORFs from the analyzed genomes. According to the complexity of the adjacent ORF rearrangements, two groups were created; the OC group was characterized by the presence of two or more ORFs in the same DNA strand until the occurrence of ORFs in the opposite strand, and the monocistronic (mC) group represented single ORFs. Differences in the annotation were evaluated by comparing the protein sequences from these three genomes using the NCBI/BLASTP program. The OC groups predicted for *M. flocculare* and *M. hyorhinis* were compared with the OC organization found in *M. hyopneumoniae*[30]. Moreover, comparative analyses were also performed between *M. hyorhinis* and *M. flocculare* to predict OC organization. The *M. flocculare* analysis was performed with each contig sequence, while the *M. hyorhinis* analysis was performed with the whole genome sequence.

### Analysis of surface-protein-encoding CDSs

For the surface protein predictions, all of the translated CDS entries from the *M. flocculare* genome and from the *M. hyopneumoniae* 7448 and *M. hyorhinis* HUB-1 genomes (NC_007332 and NC_014448 entries, respectively) were analyzed using the default parameters by the following software programs: SVMtm Transmembrane Domain Predictor [95], TMHMM Server v. 2.0 [96], SCAMPI [97], and PSORTb [98]. The first three programs predicted the presence of transmembrane (TM) domains, and the fourth was used for to predict subcellular localization (i.e., cytoplasmic vs. membrane). The first three independent TM predictions were merged, and the CDSs were considered as ‘surface protein encoding’ when they were predicted as such by at least two of the TM-predicting programs. The CDSs that were predicted to encode surface proteins by only one of the TM-predicting programs and/or by PSORTb were additionally analyzed by HMM-TM [99], and when a previously described ortholog was identified, based on the published literature. Those CDSs that were able to predict surface localization either by HMM-TM or based on the literature were also included in the list of predicted surface proteins encoded by each genome. After orthologs were identified by reciprocal pairwise comparisons between *M. flocculare*, *M. hyopneumoniae* 7448 and *M. hyorhinis* HUB-1 (see below), the CDSs predicted to encode surface proteins just for one species (by the aforementioned criteria) had their corresponding orthologs (when existent) included in the list of surface-protein-encoding CDSs for the other two species. The clusters of orthologous group (COG) functional classification of *M. flocculare* predicted surface proteins using NCBI COGnitor [100], and those of *M. hyopneumoniae* 7448 and *M. hyorhinis* HUB-1 predicted surface proteins obtained from NCBI.

For the ortholog and paralog identifications, bidirectional local BLAST searches [88] were performed between the *M. flocculare* predicted surface protein encoding CDSs and all of the CDSs from the other two species using the BioEdit Sequence Alignment Editor [101]. The ortholog and paralog definitions were based on the best hits from TBLASTX with the following parameters: cut-off e-value thresholds of 10^-20^, 10^-10^ or 10^-6^; manual inspection of query coverage, identity and similarity scores; and, if required, consideration of peculiarities of specific gene families.

### Phylogenomic analyses

*M. flocculare* (ATCC 27716) was used as query organism. We began with all 582 genes identified in this strain and performed a BLAST search against the gene set of all other 32 organisms (Additional file 34). To select putative orthologs for all of the *M. flocculare* genes, we performed a BLAST search between this queried gene set and the individual genomes of the organisms previously cited; we retrieved the single best hit from each genome (BLAST cut-off 10). Multi-FASTA files were created containing ortholog gene sets for each query gene in the *M. flocculare* strain. Only gene sets containing at least one representative in each genome were selected for further phylogenomic analysis (i.e., we only evaluated files containing 32 sequences for each analyzed genome).

Multi-FASTA putative ortholog files containing the best representative of each *M. flocculare* deduced protein sequence for all of the analyzed swine mycoplasmas were used as the input for multiple alignments using the CLUSTALw algorithm with default parameters.

The SCaFos software program [102] was used to facilitate the concatenation of the 179 alignment files. Phylogenies of 32 concatenated, deduced amino acid sequences were estimated by the NJ [103] and MP [104] methods in the Molecular Evolutionary Genetics Analysis (MEGA) program version 5.05 [105]. For the NJ method, the evolutionary distances were computed using the p-distance and the Poisson-corrected amino acids distance; both were presented in units of amino acid differences per site. The complete and pairwise deletion of gaps or missing data were implemented with the datasets containing 49,751 and 104,097 positions, respectively. The bootstrap test of the phylogeny was performed using 1,000 repetitions. The MP tree was obtained using the close-neighbor-interchange algorithm with search level 1, in which the initial trees were obtained by random addition of sequences (10 replicates). The different gap treatments were tested considering the complete deletion, partial deletion, and all sites included. The bootstrap test was implemented using 500 replicates. The TreeView software program [106] was used to visualize the resulting phylogenies.

### Paralog analysis

Selected gene clusters of paralogs were subjected to a phylogenetic analysis. BLAST searches were first conducted for each gene using a 10^-6^ e-value cutoff; all of the sequences were subsequently aligned using COBALT [107]. Distance and parsimony methods in the MEGA 5 software program were applied to identify the evolutionary scenario for each paralog cluster. A bootstrap test was additionally performed with 1,000 replications.

### Horizontal gene transfer

Horizontal gene transfer (HGT) was analyzed with Tree and Reticulogram reconstruction (T-Rex) [108] using the bipartition dissimilarity as the optimization criteria in the HGT detection algorithm [109]. The program infers an optimal (i.e., minimum-cost) scenario of horizontal gene transfers reconciling a given pair of species and gene trees. All of the gene trees were obtained through the distance method implemented in the MEGA 5 software program using p-distance, pairwise deletion of gaps, and bootstrap test of phylogeny with 500 replications. A bootstrap cutoff of 75% was applied to accept the HGT events. In total, 179 genes were subjected to phylogenetic and HGT analyses.

## Abbreviations

CDS: Coding DNA sequence; COG: Clusters of orthologous groups; HGT: Horizontal gene transfer; ORF: Open read frames; OC: ORF cluster.

## Competing interests

The authors declare they have no competing interests.

## Authors’ contributions

FMS performed the genomes organization analyses and comparative analyses of genomes, participated in the interpretation of the results and in the writing of the manuscript. CET, FP and MMBF carried out the phylogenomics and phylogenetics analyses and in the interpretation of the results. LGPA and RS carried out the assemblies genomes and participated in the comparative analyses of genomes. VGV, LR and TG participated in the surface proteins *in silico* analyses. ISS, HBF, ATV and AZ conceived, designed and coordinated the study, participated in the interpretation of the results and in the writing of the manuscript. All authors read and approved the final manuscript.

## Supplementary Material

Additional file 1 **OCs organization in *M. flocculare *genome.** Click here for file

Additional file 2 **OCs in *M. hyorhinis *genome.** Click here for file

Additional file 3 Monocistronic group (mC). Click here for file

Additional file 4 **OC organization similarities in the *M. hyorhinis *and *M. flocculare* genomes in relationship of *M. hyopneumoniae *OC organization.** Click here for file

Additional file 5 Monocistronic ORFs presents in the MHP, MHR and MFL genomes. Click here for file

Additional file 6 **A. Exclusive OCs of *M. hyopneumoniae *genome. **6B. Exclusive OCs of *M. flocculare *genome. 6C. Conserved Ocs among the three mycoplasmas species. 6D. Exclusive OCs of *M. hyorhinis* genome. Click here for file

Additional file 7 **A. *M. flocculare *contigs with conservation regions. **B. *M. flocculare *contigs with inversion regions. Click here for file

Additional file 8 **Venn diagram of the predicted surface protein sets from *M. flocculare*, *M. hyopneumoniae *7448 and *M. hyorhinis *HUB-1.** Click here for file

Additional file 9 **COG functional classifications of the predicted surface protein sets from *M. flocculare *(A), *M. hyopneumoniae *7448 (B) and *M. hyorhinis *HUB-1 (C).** Click here for file

Additional file 10 ** List of predicted surface proteins of*M. flocculare *(A), *M. hyopneumoniae *7448 (B), and *M. hyorhinis *HUB-1 (C). **B: List of predicted surface proteins of *M. flocculare* (A), *M. hyopneumoniae *7448 (B), and *M. hyorhinis *HUB-1 (C). List of predicted surface proteins of M. flocculare (A), *M. hyopneumoniae *7448 (B), and *M. hyorhinis *HUB-1 (C). Click here for file

Additional file 11 **A. List of predicted surface proteins of *M. flocculare *not shared with *M. hyopneumoniae *7448 and *M. hyorhinis *HUB_1. **B. List of predicted surface proteins of *M. hyopneumoniae* 7448 not shared with *M. flocculare *and *M. hyorhinis *HUB-1. C. List of predicted surface proteins of *M. hyorhinis* HUB-1 not shared with *M. flocculare* and *M. hyopneumoniae* 7448. Click here for file

Additional file 12 **List of ortholog surface protein coding CDSs between *M. flocculare*, *M. hyopneumoniae *7448 and *M. hyorhinis *HUB-1.** Click here for file

Additional file 13 Summary of adhesins associated to pathogenicity and Genome organization comparison. Click here for file

Additional file 14 ***M. flocculare*, *M. hyopneumoniae *and *M. hyorhynis *P97 and P97-like adhesin orthologs and paralogs.** Click here for file

Additional file 15 **Evolutionary history of mycoplasmas obtained through a phylogenomic approach.** The Neighbor-Joining method, using p-distance to compute the evolutionary distances and complete deletion of gaps was implemented by MEGA 5 software. The percentage of replicate trees in which the associated taxa clustered together in the bootstrap test (1,500 replicates) are shown next to the branches. *S. pyogenes *was used as outgroup. Click here for file

Additional file 16 **Evolutionary history of mycoplasmas obtained through a phylogenomic approach.** The Neighbor-Joining method, using Poisson correction to compute the evolutionary distances and complete deletion of gaps was implemented by MEGA 5 software. The percentage of replicate trees in which the associated taxa clustered together in the bootstrap test (1,500 replicates) are shown next to the branches. *S. pyogenes* was used as outgroup. Click here for file

Additional file 17 **Evolutionary history of mycoplasmas obtained through a phylogenomic approach.** The Neighbor-Joining method was the same description of the Additional file 16. Click here for file

Additional file 18 **Evolutionary history of mycoplasmas obtained through a phylogenomic approach.** The Maximum Parsimony method using the close-neighbor-interchange algorithm, and the complete deletion of gaps was implemented by MEGA 5 software. The percentage of replicate trees in which the associated taxa clustered together in the bootstrap test (500 replicates) are shown next to the branches. *S. pyogenes* was used as outgroup. Click here for file

Additional file 19 **Evolutionary history of DNA methylases from mycoplasmas obtained through a phylogenetic analysis. **The Neighbor-Joining method, using p-distance to compute the evolutionary distances and complete deletion of gaps was implemented by MEGA 5 software. The percentage of replicate trees in which the associated taxa clustered together in the bootstrap test (1,000 replicates) is shown next to the branches. Click here for file

Additional file 20 **Evolutionary history of ATP synthases from mycoplasmas obtained through a phylogenetic analysis. **The Neighbor-Joining method was the same description of the Additional file 19. Click here for file

Additional file 21 **Evolutionary history of ribulose-phosphate-3epimerases from mycoplasmas obtained through a phylogenetic analysis. **The Neighbor-Joining method was the same description of the Additional file 19. Click here for file

Additional file 22 **Evolutionary history of oligoendopeptidases from mycoplasmas obtained through a phylogenetic analysis. **The Neighbor-Joining method was the same description of the Additional file 16. The percentage of replicate trees in which the associated taxa clustered together in the bootstrap test (1,000 replicates) is shown next to the branches. (PDF 9 kb) Click here for file

Additional file 23 **Evolutionary history of single-strand binding proteins from mycoplasmas obtained through a phylogenetic analysis. **The Neighbor-Joining method was the same description of the Additional file 19. Click here for file

Additional file 24 **Evolutionary history of fructose-bisphosphate aldolase proteins from mycoplasmas obtained through a phylogenetic analysis. **The Neighbor-Joining method was the same description of the Additional file 19. Click here for file

Additional file 25 **Evolutionary history of dihydrolipoamide dehydrogenase proteins from mycoplasmas obtained through a phylogenetic analysis. **The Neighbor-Joining method was the same description of the Additional file 19. Click here for file

Additional file 26 **Evolutionary history of glucose-6-phosphate-isomerase proteins from mycoplasmas obtained through a phylogenetic analysis. **The Neighbor-Joining method was the same description of the Additional file 19. Click here for file

Additional file 27: **Evolutionary history of lypoate protein ligases from mycoplasmas obtained through a phylogenetic analysis. **The Neighbor-Joining method was the same description of the Additional file 19. Click here for file

Additional file 28 **Evolutionary history of acyl carrier phosphodiesterases from mycoplasmas obtained through a phylogenetic analysis. **The Neighbor-Joining method was the same description of the Additional file 19. Click here for file

Additional file 29 **Evolutionary history of lactate dehydrogenases from mycoplasmas obtained through a phylogenetic analysis. **The Neighbor-Joining method was the same description of the Additional file 19. Click here for file

Additional file 30 **Evolutionary history of membrane nucleases from mycoplasmas obtained through a phylogenetic analysis. **The Neighbor-Joining method was the same description of the Additional file 19. Click here for file

Additional file 31 **Evolutionary history of TRSE-like proteins from mycoplasmas obtained through a phylogenetic analysis. **The Neighbor-Joining method was the same description of the Additional file 19. Click here for file

Additional file 32 **Evolutionary history of P97 proteins from mycoplasmas obtained through a phylogenetic analysis. **The Neighbor-Joining method was the same description of the Additional file 19. Click here for file

Additional file 33 HGT events in mycoplasmas species. Click here for file

Additional file 34 Bacterial strains used in the phylogenomic analyses. Click here for file

## References

[B1] Razin S Yogev D Naot Y Molecular biology and pathogenicity of mycoplasmas Microbiol Mol Biol Rev 1998 62 1094 1156 9841667 10.1128/mmbr.62.4.1094-1156.1998 PMC98941

[B2] Rottem S Interaction of mycoplasmas with host cells Physiol Rev 2003 83 417 432 12663864 10.1152/physrev.00030.2002

[B3] Zhang S Lo SC Effect of mycoplasmas on apoptosis of 32D cells is species-dependent Current Microbiol 2007 54 388 395 10.1007/s00284-006-0491-x 17486403

[B4] Vogl G Plaickner A Szathmary S Stipkovits L Rosengarten R Szostak MP *Mycoplasma gallisepticum* invades chicken erythrocytes during infection Infect Immun 2008 76 71 77 10.1128/IAI.00871-07 17954728 PMC2223666

[B5] Dusanic D Bercic RL Cizelj I Salmic S Narat M Bencina D *Mycoplasma synoviae* invades non-phagocytic chicken cells in vitro Vet Microbiol 2009 138 114 119 10.1016/j.vetmic.2009.02.014 19321273

[B6] Groebel K Hoelzle K Wittenbrink MM Ziegler U Hoelzle LE *Mycoplasma suis* invades porcine erythrocytes Infect Immun 2009 77 suppl 2 576 584 19015255 10.1128/IAI.00773-08 PMC2632055

[B7] Song Z Li Y Liu Y Xin J Zou X Sun W α-enolase, an adhesion-related factor of *Mycoplasma bovis* PLoS One 2012 7 suppl 6 e38836 22719960 10.1371/journal.pone.0038836 PMC3374825

[B8] Hopfe M Deenen R Degrandi D Köhrer K Henrich B Host cell responses to persistent mycoplasmas - different stages in infection of HeLa cells with *Mycoplasma hominis* PLoS One 2013 8 suppl 1 e54219 23326599 10.1371/journal.pone.0054219 PMC3543322

[B9] Mare CJ Switzer WP *Mycoplasma hyopenumoniae*, a causative agent of virus pig pneumonia Vet Med 1965 60 841 846 14323369

[B10] Meyling A Friis NF Serological identification of a new porcine mycoplasma species, *Mycoplasma flocculare* Acta Vet Scand 1972 13 287 289 4675802 10.1186/BF03548589 PMC8561508

[B11] Rose DL Tully JG Wittler RG Taxonomy of some swine mycoplasmas: *Mycoplasma suipneumoniae* Goodwin et al. 1965, A later, objective synonym of *Mycoplasma hyopneumoniae* mare and Switzer 1965, and the status of *Mycoplasma flocculare* meyling and friis 1972 Int J Syst Evol Microbiol 1979 29 83 91

[B12] Stemke GW Laigret F Grau O Bové JM Phylogenetic relationships of three porcine mycoplasmas, *mycoplasma hyopneumoniae*, *mycoplasma flocculare* and *mycoplasma hyorhinis*, and complete 16S rRNA sequence of *M. Flocculare* Int J Syst Bacteriol 1992 42 220 225 10.1099/00207713-42-2-220 1374621

[B13] Friis NF Feenstra AA *Mycoplasma hyorhinis* in the etiology of serositis among piglets Acta Vet Scand 1994 35 93 98 8209825 10.1186/BF03548359 PMC8101397

[B14] Kobisch M Friis NF Swine mycoplasmoses Rev Sci Tech Oie 1996 15 1569 1605 10.20506/rst.15.4.983 9190026

[B15] Young TF Thacker EL Erickson BZ Ross RF A tissue culture system to study respiratory ciliary epithelial adherence of selected swine mycoplasmas Vet Microbiol 2000 71 269 279 10.1016/S0378-1135(99)00176-5 10703709

[B16] Boye M Jensen TK Ahrens P Hagedorn-Olsen T Friis NF In situ hybridisation for identification and differentiation of *Mycoplasma hyopneumoniae*, *Mycoplasma hyosynoviae* and *Mycoplasma hyorhinis* in formalin-fixed porcine tissue sections Acta Path Micro Im 2001 109 656 664 10.1034/j.1600-0463.2001.d01-129.x 11890568

[B17] Huang S Li JY Wu J Meng L Shou CC Mycoplasma infections and different human carcinomas World Gastroentero 2001 7 266 269 10.3748/wjg.v7.i2.266 PMC4723534 11819772

[B18] Yang H Qu L Ma H Chen L Liu W Liu C Meng L Wu J Shou C *Mycoplasma hyorhinis* infection in gastric carcinoma and its effects on the malignant phenotypes of gastric cancer cells BMC Gastroenterol 2010 10 132 10.1186/1471-230X-10-132 21062494 PMC2993648

[B19] Blank WA Stemke GW A physical and genetic map of the *Mycoplasma flocculare* ATCC 27716 chromosome reveals large genomic inversions when compared with that of *Mycoplasma hyopneumoniae* strain J Int J Syst Evol Micr 2001 51 1395 1399 10.1099/00207713-51-4-1395 11491338

[B20] Minion FC Lefkowitz EL Madsen ML Cleary BJ Swartzell SM Mahairas GG The genome sequence of strain 232, the agent of swine mycoplasmosis J Bacteriol 2004 186 7123 7133 10.1128/JB.186.21.7123-7133.2004 15489423 PMC523201

[B21] Vasconcelos ATR Ferreira HB Bizarro CV Bonatto SL Carvalho MO Pinto PM Almeida DF Almeida LGP Almeida R Alves-Filho L Assunção EN Azevedo VAC Bogo MR Brigido MM Brocchi M Burity HA Camargo AA Camargo SS Carepo MS Carraro DM de Mattos Cascardo JC Castro LA Cavalcanti G Chemale G Collevatti RG Cunha CW Dallagiovanna B Dambrós BP Dellagostin OA Falcão C Swine and poultry pathogens: the complete genome sequences of two strains of *mycoplasma hyopneumoniae* and a strain of *mycoplasma synoviae* J Bacteriol 2005 187 5568 5577 10.1128/JB.187.16.5568-5577.2005 16077101 PMC1196056

[B22] Liu W Fang L Li S Li Q Zhou Z Feng Z Luo R Shao G Wang L Chen H Xiao S Complete genome sequence of *Mycoplasma hyorhinis* strain HUB-1 J Bacteriol 2010 192 suppl 21 5844 5845 20802032 10.1128/JB.00946-10 PMC2953675

[B23] Liu W Feng Z Fang L Zhou Z Li Q Li S Luo R Wang L Chen H Shao G Xiao S Complete genome sequence of *Mycoplasma hyopneumoniae* strain 168 J Bacteriol 2011 193 suppl 4 1016 1017 21148737 10.1128/JB.01305-10 PMC3028675

[B24] Guimaraes AM Santos AP SanMiguel P Walter T Timenetsky J Messick JB Complete genome sequence of *Mycoplasma suis* and insights into its biology and adaption to an erythrocyte niche PLoS One 2011 6 suppl 5 e19574 21573007 10.1371/journal.pone.0019574 PMC3091866

[B25] Oehlerking J Kube M Felder KM Matter D Wittenbrink MM Schwarzenbach S Kramer MM Hoelzle K Hoelzle LE Complete genome sequence of the hemotrophic *Mycoplasma suis* strain KI3806 J Bacteriol 2011 193 suppl 9 2369 2370 21398558 10.1128/JB.00187-11 PMC3133081

[B26] Kornspan JD Lysnyansky I Kahan T Herrmann R Rottem S Nir-Paz R Genome analysis of a *Mycoplasma hyorhinis* strain derived from a primary human melanoma cell line J Bacteriol 2011 193 suppl17 4543 4544 21705582 10.1128/JB.05505-11 PMC3165497

[B27] Calcutt MJ Foecking MF Rosales RS Ellis RJ Nicholas RA Genome sequence of *Mycoplasma hyorhinis* strain GDL-1 J Bacteriol 2012 194 suppl 7 1848 22408248 10.1128/JB.00033-12 PMC3302467

[B28] Goodison S Urquidi V Kumar D Reyes L Rosser CJ Complete genome sequence of *Mycoplasma hyorhinis* strain SK76 Genome Announc 2013 1 suppl 1 e00101 e00112 23405353 10.1128/genomeA.00101-12 PMC3569356

[B29] Kanehisa M Goto S KEGG: kyoto encyclopedia of genes and genomes Nucleic Acids Res 2000 28 27 30 10.1093/nar/28.1.27 10592173 PMC102409

[B30] Siqueira FM Schrank A Schrank IS *Mycoplasma hyopneumoniae* transcription unit organization: genome survey and prediction DNA Res 2011 18 413 422 10.1093/dnares/dsr028 22086999 PMC3223074

[B31] Rogozin IB Makarova KS Murvai J Czabarka E Wolf YI Tatusov RL Szekely LA Koonin EV Connected gene neighborhoods in prokaryotic genomes Nucleic Acids Res 2002 30 2212 2223 10.1093/nar/30.10.2212 12000841 PMC115289

[B32] Lathe WC Snel B Bork P Gene context conservation of a higher order than operons Trends Biochem Sci 2000 25 suppl 10 474 479 11050428 10.1016/s0968-0004(00)01663-7

[B33] Rosengarten R Wise KS Phenotypic switching in mycoplasmas: phase variation of diverse surface lipoproteins Science 1990 247 315 318 10.1126/science.1688663 1688663

[B34] Himmelreich R Plagens H Hilbert H Reiner B Herrmann R Comparative analysis of the genomes of the bacteria *Mycoplasma pneumoniae* and *Mycoplasma genitalium* Nucleic Acids Res 1997 25 701 712 10.1093/nar/25.4.701 9016618 PMC146517

[B35] Mahan MJ Roth JR Role of recBC function in formation of chromosomal rearrangements: a Two-step model for recombination Genetics 1989 121 433 443 2714635 10.1093/genetics/121.3.433 PMC1203631

[B36] Carvalho FM Fonseca MM Medeiros SB Scortecci KC Blaha CAG Agnez-Lima LF DNA repair in reduced genome: the Mycoplasma model Gene 2005 360 suppl 2 111 119 16153783 10.1016/j.gene.2005.06.012

[B37] Burgos R Wood GE Young L Glass JI Totten PA RecA mediates MgpB and MgpC phase and antigenic variation in *Mycoplasma genitalium*, but plays a minor role in DNA repair Mol Microbiol 2012 85 suppl 4 669 683 22686427 10.1111/j.1365-2958.2012.08130.x PMC3418420

[B38] Burnett TA Dinkla K Rohde M Chhatwal GS Uphoff C Srivastava M Cordwell SJ Geary S Liao X Minion FC Walker MJ Djordjevic SP P159 is a proteolytically processed, surface adhesin of *Mycoplasma hyopneumoniae*: defined domains of P159 bind heparin and promote adherence to eukaryote cells Mol Microbiol 2006 60 669 686 10.1111/j.1365-2958.2006.05139.x 16629669

[B39] Wilton J Jenkins C Cordwell SJ Falconer L Minion FC Oneal DC Djordjevic MA Connolly A Barchia I Walker MJ Djordjevic SP Mhp493 (P216) is a proteolytically processed, cilium and heparin binding protein of *Mycoplasma hyopneumoniae* Mol Microbiol 2009 71 566 582 10.1111/j.1365-2958.2008.06546.x 19040640

[B40] Seymour LM Deutscher AT Jenkins C Kuit TA Falconer L Minion FC Crossett B Padula M Dixon NE Djordjevic SP Walker MJ A processed multidomain *mycoplasma hyopneumoniae* adhesin binds fibronectin, plasminogen, and swine respiratory cilia J Biol Chem 2010 285 33971 33978 10.1074/jbc.M110.104463 20813843 PMC2962497

[B41] Deutscher AT Jenkins C Minion FC Seymour LM Padula MP Dixon NE Walker MJ Djordjevic SP Repeat regions R1 and R2 in the P97 paralogue Mhp271 of Mycoplasma hyopneumoniae bind heparin, fibronectin and porcine cilia Mol Microbiol 2010 78 444 458 10.1111/j.1365-2958.2010.07345.x 20879998

[B42] Seymour LM Falconer L Deutscher AT Minion FC Padula MP Dixon NE Djordjevic SP Walker MJ Mhp107 Is a member of the multifunctional adhesin family of *Mycoplasma hyopneumoniae* J Biol Chem 2011 286 suppl 12 10097 10104 21245147 10.1074/jbc.M110.208140 PMC3060461

[B43] Seymour LM Jenkins C Deutscher AT Raymond BBA Padula MP Tacchi JL Bogema DR Eamens GJ Woolley LK Dixon NE Walker MJ Djordjevic SP Mhp182 (P102) binds fibronectin and contributes to the recruitment of plasmin(ogen) to the Mycoplasma hyopneumoniae cell surface Cell Microbiol 2012 14 suppl 1 81 94 21951786 10.1111/j.1462-5822.2011.01702.x

[B44] Bogema DR Deutscher AT Woolley LK Seymour LM Raymond BB Tacchi JL Padula MP Dixon NE Minion FC Jenkins C Walker MJ Djordjevic SP Characterization of cleavage events in the multifunctional cilium adhesin Mhp684 (P146) reveals a mechanism by which *Mycoplasma hyopneumoniae* regulates surface topography MBio 2012 3 suppl 2 e00282 11 22493032 10.1128/mBio.00282-11 PMC3322551

[B45] Zielinski GC Ross RF Effect on growth in cell cultures and strain on virulence of *Mycoplasma hyopneumoniae* for swine Am J Vet Res 1990 51 344 348 2180349

[B46] Pinto P Klein C Zaha A Ferreira H Comparative proteomic analysis of pathogenic and non-pathogenic strains from the swine pathogen *Mycoplasma hyopneumoniae* Proteome Science 2009 7 45 10.1186/1477-5956-7-45 20025764 PMC2804596

[B47] Castro LAD Pedroso TR Kuchiishi SS Ramenzoni M Kich JD Zaha A Vainstein MH Ferreira HB Variable number of tandem aminoacid repeats in adhesion-related CDS products in *Mycoplasma hyopneumoniae* strains Vet Microbiol 2006 116 258 269 10.1016/j.vetmic.2006.04.022 16730926

[B48] Yogev D Watson-McKown R Rosengarten R Im J Wise KS Increased structural and combinatorial diversity in an extended family of genes encoding Vlp surface proteins of *Mycoplasma hyorhinis* J Bacteriol 1995 177 5636 5643 7559353 10.1128/jb.177.19.5636-5643.1995 PMC177375

[B49] Citti C Kim MF Wise KS Elongated versions of Vlp surface lipoproteins protect *Mycoplasma hyorhinis* escape variants from growth-inhibiting host antibodies Infect Immun 1997 65 suppl 5 1773 1785 9125561 10.1128/iai.65.5.1773-1785.1997 PMC175216

[B50] Citti C Nouvel LX Baranowski E Phase and antigenic variation in mycoplasmas Future Microbiol 2010 5 suppl 7 1073 1085 20632806 10.2217/fmb.10.71

[B51] Hickman AB Chandler M Dyda F Integrating prokaryotes and eukaryotes: DNA transposases in light of structure Crit Rev Biochem Mol Biol 2010 45 50 69 10.3109/10409230903505596 20067338 PMC3107681

[B52] Westberg J Persson A Holmberg A Goesmann A Lundeberg J Johansson K-E Pettersson B Uhlén M The genome sequence of *mycoplasma mycoides* subsp. *Mycoides* SC type strain PG1T, the causative agent of contagious bovine pleuropneumonia (CBPP) Genome Res 2004 14 221 227 10.1101/gr.1673304 14762060 PMC327097

[B53] Li Y Zheng H Liu Y Jiang Y Xin J Chen W Song Z The complete genome sequence of *mycoplasma bovis* strain hubei-1 PLoS One 2011 6 e20999 10.1371/journal.pone.0020999 21731639 PMC3120828

[B54] Peters IR Helps CR McAuliffe L Neimark H Lappin MR Gruffydd-Jones TJ Day MJ Hoelzle LE Willi B Meli M Hofmann-Lehmann R Tasker S RNase P RNA gene (rnpB) phylogeny of Hemoplasmas and other *Mycoplasma* species J Clin Microbiol 2008 46 1873 1877 10.1128/JCM.01859-07 18337389 PMC2395117

[B55] Volokhov DV Simonyan V Davidson MK Chizhikov VE RNA polymerase beta subunit (rpoB) gene and the 16S–23S rRNA intergenic transcribed spacer region (ITS) as complementary molecular markers in addition to the 16S rRNA gene for phylogenetic analysis and identification of the species of the family Mycoplasmataceae Mol Phylogen Evol 2011 62 515 528 10.1016/j.ympev.2011.11.002 22115576

[B56] Marinus MG Casadesus J Roles of DNA adenine methylation in host- pathogen interactions: mismatch repair, transcriptional regulation, and more FEMS Microbiol Rev 2009 33 488 503 10.1111/j.1574-6976.2008.00159.x 19175412 PMC2941194

[B57] Kumar R Mukhopadhyay AK Ghosh P Rao DN Comparative transcriptomics of H. pylori strains AM5, SS1, and their hpyAVIBM deletion mutants: possible roles of cytosine methylation PLoS One 2012 7 e42303 10.1371/journal.pone.0042303 22879937 PMC3411764

[B58] Reisenauer A Kahng LS McCollum S Shapiro L Bacterial DNA methylation: a cell cycle regulator J Bacteriol 1999 181 5135 5139 10464180 10.1128/jb.181.17.5135-5139.1999 PMC94015

[B59] Wion D Casadesus J N6-methyl-adenine: an epigenetic signal for DNA- protein interactions Nat Rev Microbiol 2006 4 183 192 10.1038/nrmicro1350 16489347 PMC2755769

[B60] Fälker S Schmidt MA Heusipp G DNA adenine methylation and bacterial pathogenesis Int J Med Microbiol 2007 297 1 7 17126598 10.1016/j.ijmm.2006.10.002

[B61] Schwartz D Quinn TJ Thorne PS Sayeed S Yi A-K Krieg AM CpG motifs in bacterial DNA cause inflammation in the lower respiratory tract J Clin Invest 1997 100 68 73 10.1172/JCI119523 9202058 PMC508166

[B62] Butler JE Young ND Lovley DR Evolution of electron transfer out of the cell: comparative genomics of six *Geobacter* genomes BMC Genomics 2010 11 40 10.1186/1471-2164-11-40 20078895 PMC2825233

[B63] Rogers MB Watkins RF Harper JT Durnford DG Gray MW Keeling PJ A complex and punctate distribution of three eukaryotic genes derived by lateral gene transfer BMC Evol Biol 2007 7 89 10.1186/1471-2148-7-89 17562012 PMC1920508

[B64] Grauvogel C Brinkmann H Petersen J Evolution of the glucose-6-phosphate isomerase: the plasticity of primary metabolism in photosynthetic eukaryotes Mol Biol Evol 2007 24 1611 1621 10.1093/molbev/msm075 17443012

[B65] Thomas J Cronan JE The enigmatic acyl carrier protein phosphodiesterase of *Escherichia coli* J Biol Chem 2005 280 34675 34683 10.1074/jbc.M505736200 16107329

[B66] Li WH Yang J Gu X Expression divergence between duplicate genes Trends Genet 2005 21 602 607 10.1016/j.tig.2005.08.006 16140417

[B67] Thomas PD Wood V Mungall CJ Lewis SE Blake JA On the use of gene ontology annotations to assess functional similarity among orthologs and paralogs: a short report PLoS Comput Biol 2012 8 e1002386 10.1371/journal.pcbi.1002386 22359495 PMC3280971

[B68] Henderson B Martin A Bacterial virulence in the moonlight: multitasking bacterial moonlighting proteins Are virulence determinants in infectious disease Infect Immun 2011 79 suppl 9 3476 3491 21646455 10.1128/IAI.00179-11 PMC3165470

[B69] Chen LL Chung WC Lin CP Kuo CH Comparative analysis of gene content evolution in phytoplasmas and mycoplasmas PLoS One 2012 7 suppl 3 e34407 22479625 10.1371/journal.pone.0034407 PMC3313985

[B70] Xu G Guo C Shan H Kong H Divergence of duplicate genes in exon-intron structure Proc Natl Acad Sci 2012 109 1187 1192 10.1073/pnas.1109047109 22232673 PMC3268293

[B71] Ochmann H Lawrence JG Groisman EA Lateral gene transfer and the nature of bacterial innovation Nature 2000 405 299 304 10.1038/35012500 10830951

[B72] Philippe H Douady CJ Horizontal gene transfer and phylogenetics Curr Opin Microbiol 2003 6 498 505 10.1016/j.mib.2003.09.008 14572543

[B73] Pollack JD The necessity of combining genomic and enzymatic data to infer metabolic function and pathways in the smallest bacteria: amino acid, purine and pyrimidine metabolism in Mollicutes Front Biosci 2002 7 d1762 d1781 10.2741/pollack 12133816

[B74] Matic JN Wilton JL Towers RJ Scarman AL Minion FC Walker MJ Djordjevic SP The pyruvate dehydrogenase complex of *Mycoplasma hyopneumoniae* contains a novel lipoyl domain arrangement Gene 2003 319 99 106 14597175 10.1016/s0378-1119(03)00798-4

[B75] Pereyre S Sirand-Pugnet P Beven L Charron A Renaudin H Barré A Avenaud P Jacob D Couloux A Barbe V de Daruvar A Blanchard A Bébéar C Life on arginine for *Mycoplasma hominis*: clues from its minimal genome and comparison with other human urogenital mycoplasmas PLoS Genet 2009 5 suppl 10 e1000677 19816563 10.1371/journal.pgen.1000677 PMC2751442

[B76] Alvarez RA Blaylock MW Baseman JB Surface localized glyceraldehyde-3-phosphate dehydrogenase of *Mycoplasma genitalium* binds mucin Mol Microbiol 2003 48 1417 1425 10.1046/j.1365-2958.2003.03518.x 12787366

[B77] Hoelzle K Grimm J Ritzmann M Heinritzi K Torgerson P Hamburger A Wittenbrink MM Hoelzle LE Detection of antibodies against *Mycoplasma suis* using recombinant antigens and correlation of serological results to hematological findings Clin Vaccine Immunol 2007 14 suppl 12 1616 1622 17942612 10.1128/CVI.00345-07 PMC2168379

[B78] Dumke R Hausner M Jacobs E Role of *Mycoplasma pneumoniae* glyceraldehyde-3-phosphate dehydrogenase (GAPDH) in mediating interactions with the human extracellular matrix Microbiology 2011 157 2328 2338 10.1099/mic.0.048298-0 21546586

[B79] Schreiner AS Sokoli A Felder KM Wittenbrink MM Schwarzenbach S Guhl B Hoelzle K Hoelzle LE The surface-localised a-enolase of *Mycoplasma suis* is an adhesion protein Vet Microbiol 2012 156 88 95 10.1016/j.vetmic.2011.10.010 22047714

[B80] Minion FC Jarvill-Taylor KJ Billings DE Tigges E Membrane-associated nuclease activities in mycoplasmas J Bacteriol 1993 175 7842 7847 8253673 10.1128/jb.175.24.7842-7847.1993 PMC206960

[B81] Schmidt JA Browning GF Markham PF *Mycoplasma hyopneumoniae* mhp379 is a Ca2 + -dependent, sugar-nonspecific exonuclease exposed on the cell surface J Bacteriol 2007 189 3414 3424 10.1128/JB.01835-06 17307846 PMC1855908

[B82] Bizarro CV Schuck DC Purine and pyrimidine nucleotide metabolism in Mollicutes Genet Mol Biol 2007 30 190 201 10.1590/S1415-47572007000200005

[B83] Friis NF Some recommendations concerning primary isolation of *Mycoplasma suipneumoniae* and *Mycoplasma flocculare* a survey Nordisk Vet Medicin 1975 27 337 339 1098011

[B84] Sambrook J Russell DW Molecular Cloning a Laboratory Manual 2001 New York: Cold Spring Harbor Laboratory Press

[B85] Carraro DM Camargo AA Salim AC Grivet M Vasconcelos AT Simpson AJ PCR-assisted contig extension: stepwise strategy for bacterial genome closure Biotechniques 2003 34 626 632 12661167 10.2144/03343dd05

[B86] Almeida LGP Paixão R Souza RC da Costa GC Barrientos FJA dos Santos MT de Almeida DF Vasconcelos ATR A system for automated bacterial (genome) integrated annotation-SABIA Bioinformatics 2004 20 2832 2833 10.1093/bioinformatics/bth273 15087310

[B87] Overbeek R Fonstein M D’Souza M Pusch GD Maltsev N The use of gene clusters to infer functional coupling Proc Natl Acad Sci 1999 96 2896 2901 10.1073/pnas.96.6.2896 10077608 PMC15866

[B88] Altschul S Madden T Schaffer A Zhang J Zhang Z Miller W Lipman D Gapped BLAST and PSI-BLAST: a new generation of protein database search programs Nucleic Acids Res 1997 25 3389 3402 10.1093/nar/25.17.3389 9254694 PMC146917

[B89] Apweiler R Attwood TK Bairoch A Bateman A Birney E Biswas M Bucher P Cerutti L Corpet F Croning MDR Durbin R Falquet L Fleischmann W Gouzy J Hermjakob H Hulo N Jonassen I Kahn D Kanapin A Karavidopoulou Y Lopez R Marx B Mulder NJ Oinn TM Pagni M Servant F Sigrist CJA Zdobnov EM The InterPro database, an integrated documentation resource for protein families, domains and functional sites Nucleic Acids Res 2001 29 37 40 10.1093/nar/29.1.37 11125043 PMC29841

[B90] Gardy JL Spencer C Wang K Ester M Tusnády GE Simon I Hua S deFays K Lambert C Nakai K Brinkman FSL PSORT-B: improving protein subcellular localization prediction for gram-negative bacteria Nucleic Acids Res 2003 31 3613 3617 10.1093/nar/gkg602 12824378 PMC169008

[B91] Tatusov R Fedorova N Jackson J Jacobs A Kiryutin B Koonin E Krylov D Mazumder R Mekhedov S Nikolskaya A Rao BS Smirnov S Sverdlov A Vasudevan S Wolf Y Yin J Natale D The COG database: an updated version includes eukaryotes BMC Bioinformatics 2003 4 41 10.1186/1471-2105-4-41 12969510 PMC222959

[B92] Saier MH Tran CV Barabote RD TCDB: the transporter classification database for membrane transport protein analyses and information Nucleic Acids Res 2006 34 suppl 1 D181 D186 16381841 10.1093/nar/gkj001 PMC1334385

[B93] Bairoch A Apweiler R Wu CH Barker WC Boeckmann B Ferro S Gasteiger E Huang H Lopez R Magrane M Martin MJ Natale DA O’Donovan C Redaschi N Yeh L-SL The universal protein resource (UniProt) Nucleic Acids Res 2005 33 D154 D159 15608167 10.1093/nar/gki070 PMC540024

[B94] Rutherford K Parkhill J Crook J Horsnell T Rice P Rajandream M-A Barrell B Artemis: sequence visualization and annotation Bioinformatics 2000 16 944 945 10.1093/bioinformatics/16.10.944 11120685

[B95] Yuan Z Mattick JS Teasdale RD SVMtm: support vector machines to predict transmembrane segments J Comput Chem 2004 25 632 636 10.1002/jcc.10411 14978706

[B96] Krogh A Larsson B von Heijne G Sonnhammer ELL Predicting transmembrane protein topology with a hidden markov model: application to complete genomes J Mol Biol 2001 305 567 580 10.1006/jmbi.2000.4315 11152613

[B97] Bernsel A Viklund H Falk J Lindahl E von Heijne G Elofsson A Prediction of membrane-protein topology from first principles Proc Natl Acad Sci 2008 105 7177 7181 10.1073/pnas.0711151105 18477697 PMC2438223

[B98] Yu NY Wagner JR Laird MR Melli G Rey S Lo R Dao P Sahinalp SC Ester M Foster LJ Brinkman FSL PSORTb 3.0: improved protein subcellular localization prediction with refined localization subcategories and predictive capabilities for all prokaryotes Bioinformatics 2010 26 1608 1615 10.1093/bioinformatics/btq249 20472543 PMC2887053

[B99] Bagos P Liakopoulos T Hamodrakas S Algorithms for incorporating prior topological information in HMMs: application to transmembrane proteins BMC Bioinformatics 2006 7 189 10.1186/1471-2105-7-189 16597327 PMC1523218

[B100] Tatusov RL Natale DA Garkavtsev IV Tatusova TA Shankavaram UT Rao BS Kiryutin B Galperin MY Fedorova ND Koonin EV The COG database: new developments in phylogenetic classification of proteins from complete genomes Nucleic Acids Res 2001 29 22 28 10.1093/nar/29.1.22 11125040 PMC29819

[B101] Hall TA BioEdit: a user-friendly biological sequence alignment editor and analysis program for Windows 95/98/NT Nucleic Acids Symp Ser 1999 41 95 98

[B102] Roure B Rodriguez-Ezpeleta N Philippe H SCaFoS: a tool for Selection Concatenation and Fusion of Sequences for phylogenomics. BMC Evol Biol 2007 7 S2 10.1186/1471-2148-7-S1-S2 PMC1796611 17288575

[B103] Saitou N Nei M The neighbor-joining method: a new method for reconstructing phylogenetic trees Mol Biol Evol 1987 4 406 425 3447015 10.1093/oxfordjournals.molbev.a040454

[B104] Lake JA A rate-independent technique for analysis of nucleic acid sequences: evolutionary parsimony Mol Biol Evol 1987 4 167 191 3447007 10.1093/oxfordjournals.molbev.a040433

[B105] Kumar S Nei M Dudley J Tamura K MEGA: A biologist-centric software for evolutionary analysis of DNA and protein sequences Brief Bioinform 2008 9 299 306 10.1093/bib/bbn017 18417537 PMC2562624

[B106] Page RDM Visualizing Phylogenetic Trees Using TreeView Current Protocols in Bioinformatics 2002 John Wiley & Sons, Inc 10.1002/0471250953.bi0602s01 18792942

[B107] Papadopoulos JS Agarwala R COBALT: constraint-based alignment tool for multiple protein sequences Bioinformatics 2007 23 1073 1079 10.1093/bioinformatics/btm076 17332019

[B108] Makarenkov V T-REX: reconstructing and visualizing phylogenetic trees and reticulation networks Bioinformatics 2001 17 664 668 10.1093/bioinformatics/17.7.664 11448889

[B109] Boc A Philippe H Makarenkov V Inferring and validating horizontal gene transfer events using bipartition dissimilarity Syst Biol 2010 59 195 211 10.1093/sysbio/syp103 20525630

