# Supplementary material for: New insights on the biology of swine respiratory tract mycoplasmas from a comparative genome analysis
Source: BMC Genomics. 2013 Mar 14;14:175. doi: 10.1186/1471-2164-14-175 (PMC3610235; doi:10.1186/1471-2164-14-175)
Supplement: Additional file 8 — Venn diagram of the predicted surface protein sets from M. flocculare, M. hyopneumoniae 7448 and M. hyorhinis HUB-1. [file 1471-2164-14-175-S8.pdf]

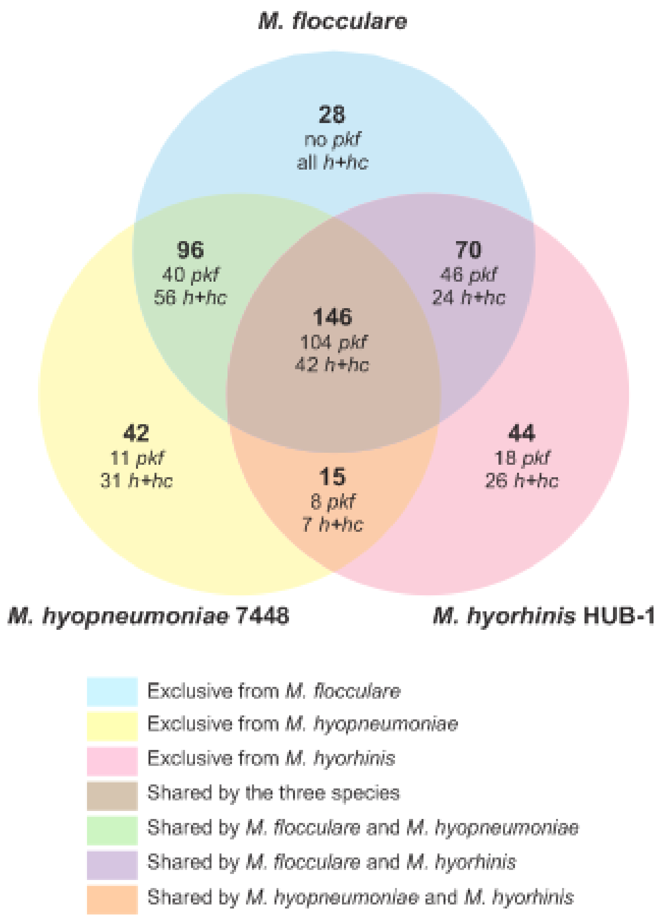


**Additional file 8. Venn diagram of the predicted surface protein sets from *M. flocculare, M. hyopneumoniae* 7448 and *M. hyorhinis* HUB-1.** Venn diagram obtained from the identification of orthologs between the predicted surface protein sets encoded by the genomes of the three analyzed species. In each area of the diagram, the total number of proteins (in bold) and the numbers of proteins with known function (*pkf*) and hypothetical + conserved hypothetical (*h+hc*) proteins are indicated. The number of proteins in a given shared category can be higher than the number of proteins in that category for one the involved species, due to the differential (between species) occurrence of paralogs. The number of proteins regarded as hypothetical or conserved hypothetical in a given shared category can be lower than the number of proteins in that category for one the involved species, due to the occurrence of an annotated ortholog.
