# Supplementary material for: New insights on the biology of swine respiratory tract mycoplasmas from a comparative genome analysis
Source: BMC Genomics. 2013 Mar 14;14:175. doi: 10.1186/1471-2164-14-175 (PMC3610235; doi:10.1186/1471-2164-14-175)
Supplement: Additional file 9 — COG functional classifications of the predicted surface protein sets from M. flocculare (A), M. hyopneumoniae 7448 (B) and M. hyorhinis HUB-1 (C). [file 1471-2164-14-175-S9.pdf]

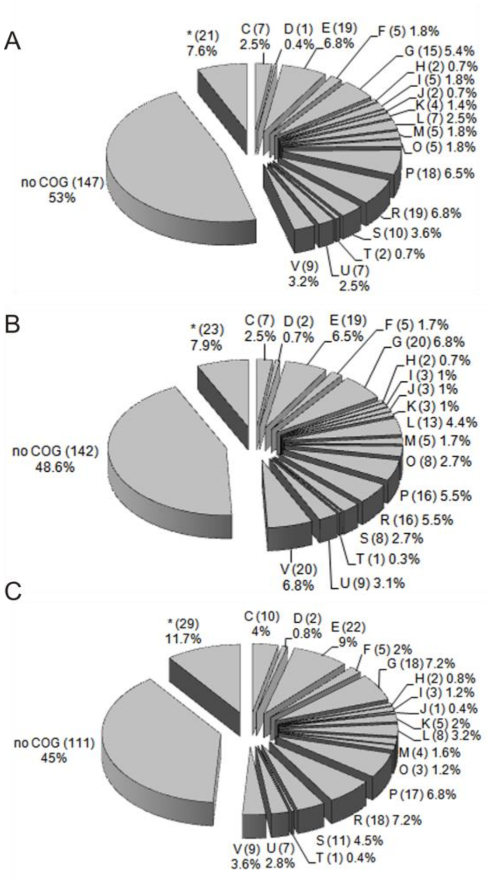

Additional file 9. COG functional classifications of the predicted surface protein sets from *M. flocculare* (A), *M. hyopneumoniae* 7448 (B) and *M. hyorhinis* HUB-1 (C). Percentages and the number (in parentheses) of proteins predicted in each functional category are indicated in the sectors of the circle. Functional categories are: (C) Energy production and conversion; (D) Cell cycle control, mitosis and meiosis; (E) Amino acid transport and metabolism; (F) Nucleotide transport and metabolism; (G) Carbohydrate transport and metabolism; (H) Coenzyme transport and metabolism; (I) Lipid transport and metabolism; (J) Translation; (K) Transcription; (L) Replication, recombination and repair; (M) Cell wall/membrane biogenesis; (O) Posttranslational modification, protein turnover, chaperones; (P) Inorganic ion transport and metabolism; (R) General function prediction only; (S) Function unknown; (T) Signal transduction mechanisms; (U) Intracellular trafficking and secretion; (V) Defense mechanisms; (no COG) Protein not related to any COG category; (*) Antigen, adhesion or lipoprotein (proteins arbitrarily classified based on their prior immunological or functional characterization according to published literature). The number of proteins in the graphic exceeds the total of predicted proteins because some of them were grouped in more than one functional category.
