# Supplementary material for: New insights on the biology of swine respiratory tract mycoplasmas from a comparative genome analysis
Source: BMC Genomics. 2013 Mar 14;14:175. doi: 10.1186/1471-2164-14-175 (PMC3610235; doi:10.1186/1471-2164-14-175)
Supplement: Additional file 14 — M. flocculare, M. hyopneumoniae and M. hyorhynis P97 and P97-like adhesin orthologs and paralogs. [file 1471-2164-14-175-S14.pdf]

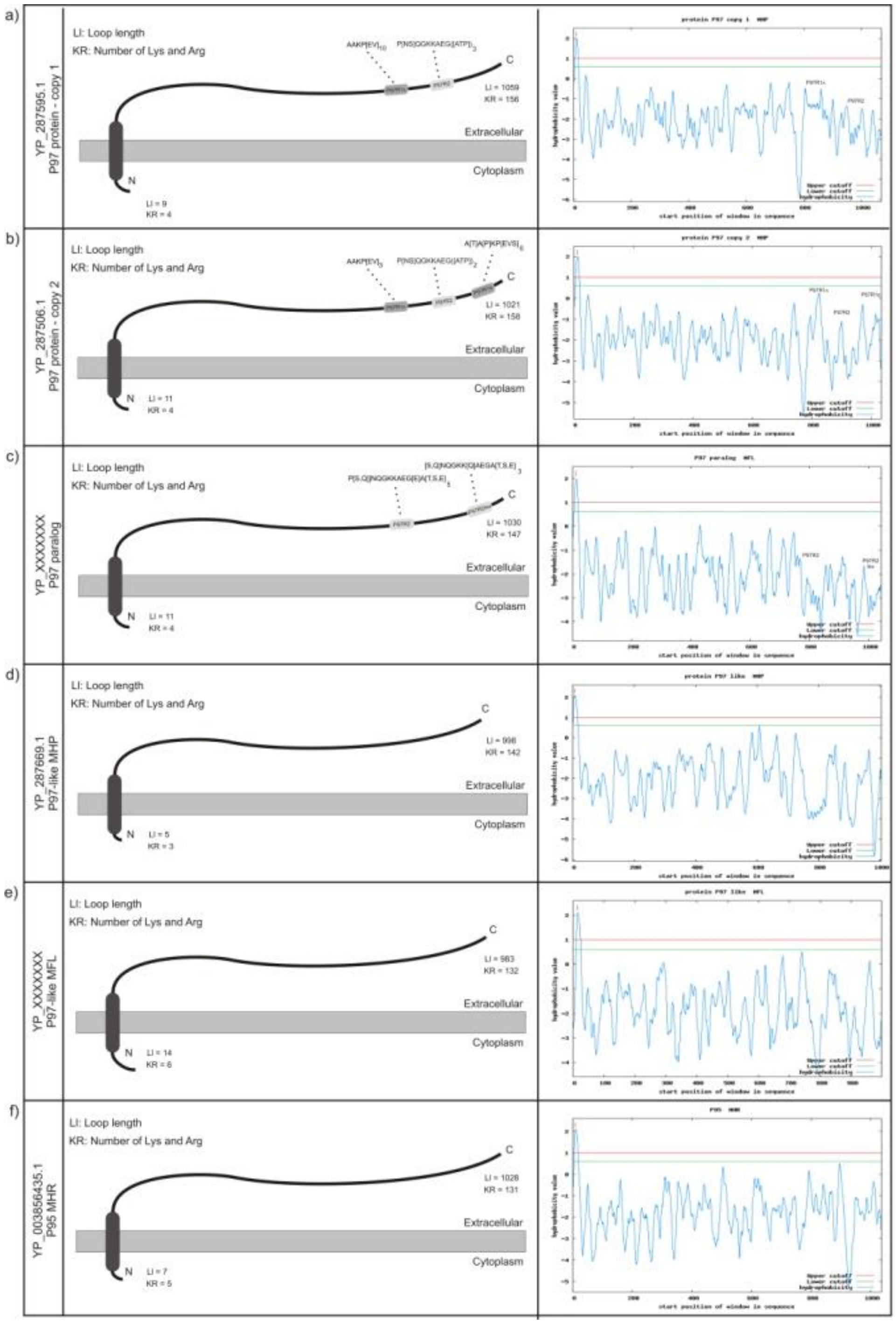

Additional file 14. ***M. flocculare*****,** ***M. hyopneumoniae*** **and** ***M. hyorhynis*** **P97 and P97-like adhesin orthologs and paralogs.** Predictions of protein topology and hydrophobicity for (a) *M. hyopneumoniae* P97 protein copy 1 (P97 C1 MHP); (b) *M. hyopneumoniae* P97 protein copy 2 from (P97 C2 MHP); (c) *M. flocculare* P97 (P97 MFL); (d) *M. hyopneumoniae* P97-like protein (P97like MHP); (e) *M. flocculare* P97-like protein (P97like MFL) (f) *M. hyorhynis* P95 protein (P95 MHR). Boxes P97R1A, P97R1B, P97R2 and P97R2LIKE indicate R1 and R2repeat regions of the P97 protein family that, at least for *M. hyopneumoniae*, play key roles in bacterial adherence. (g) Global deduced amino acid sequence alignments. Topology and hydrophobicity predictions performed using TopPred 0.01 (http://mobyle.pasteur.fr/cgi-bin/portal.py#forms::toppred); Global sequence alignments performed using ClustalW (http://www.ebi.ac.uk/Tools/msa/clustalw2/).
