# Supplementary material for: New insights on the biology of swine respiratory tract mycoplasmas from a comparative genome analysis
Source: BMC Genomics. 2013 Mar 14;14:175. doi: 10.1186/1471-2164-14-175 (PMC3610235; doi:10.1186/1471-2164-14-175)
Supplement: Additional file 17 — Evolutionary history of mycoplasmas obtained through a phylogenomic approach. The Neighbor-Joining method was the same description of the Additional file 16. [file 1471-2164-14-175-S17.pdf]

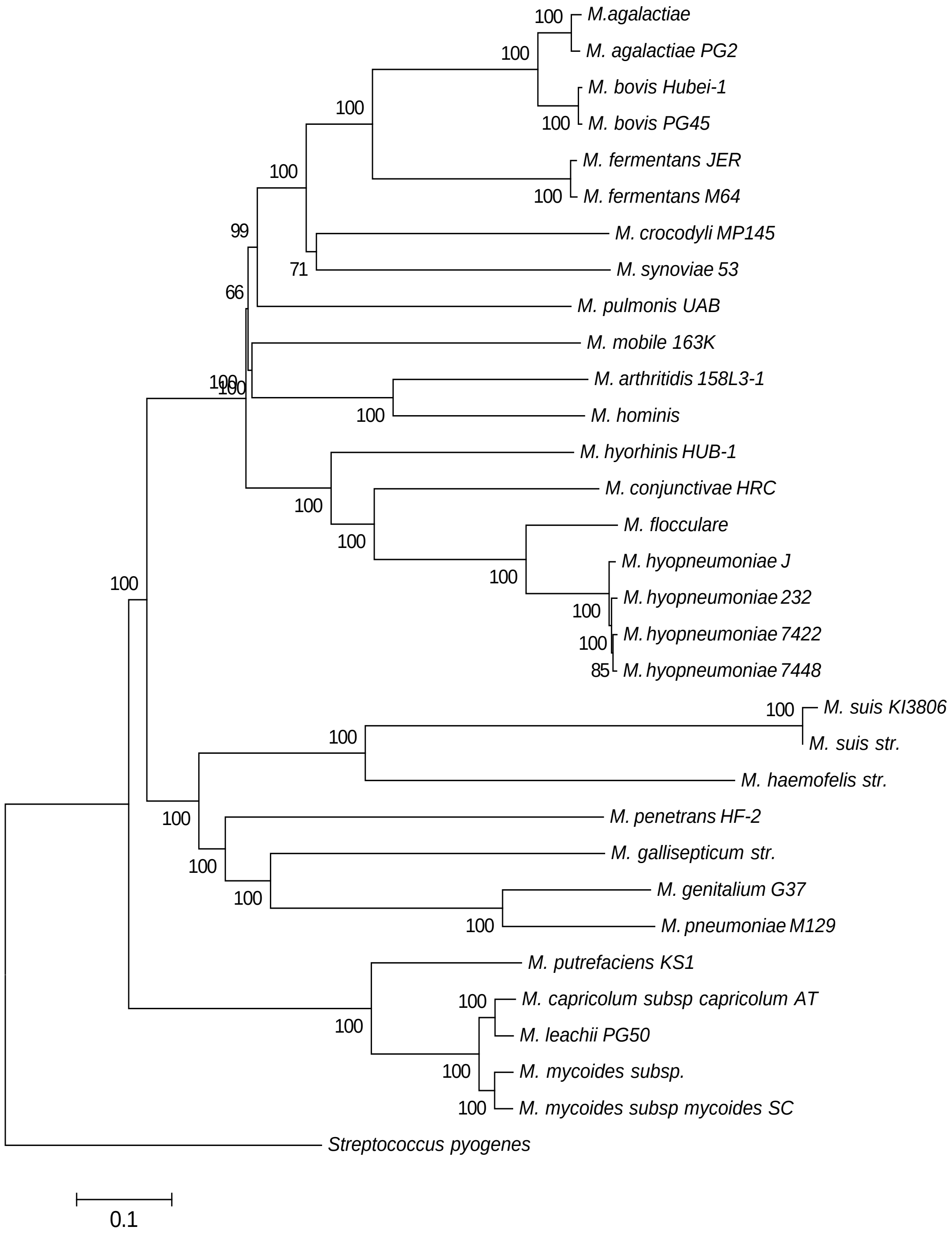

Additional file 17. **Evolutionary history of mycoplasmas obtained through a phylogenomic approach.** The Neighbor-Joining method was the same description of the Additional file 16.
