# Supplementary material for: New insights on the biology of swine respiratory tract mycoplasmas from a comparative genome analysis
Source: BMC Genomics. 2013 Mar 14;14:175. doi: 10.1186/1471-2164-14-175 (PMC3610235; doi:10.1186/1471-2164-14-175)
Supplement: Additional file 18 — Evolutionary history of mycoplasmas obtained through a phylogenomic approach. The Maximum Parsimony method using the close-neighbor-interchange algorithm, and the complete deletion of gaps was implemented by MEGA 5 software. The percentage of replicate trees in which the associated taxa clustered together in the bootstrap test (500 replicates) are shown next to the branches. S. pyogenes was used as outgroup. [file 1471-2164-14-175-S18.pdf]

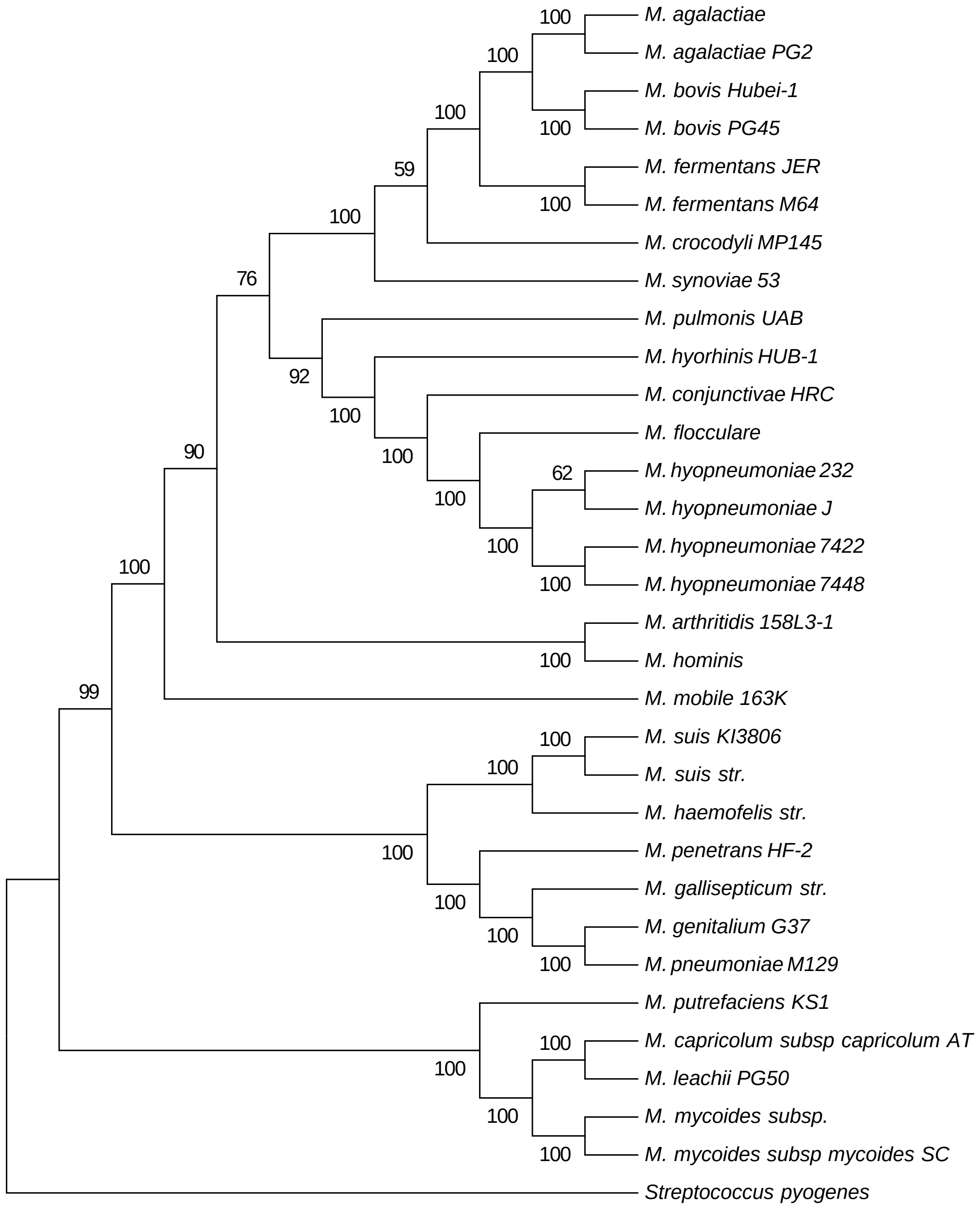


**Additional file 18**. **Evolutionary history of mycoplasmas obtained through a phylogenomic approach.** The Maximum Parsimony method using the close-neighbor-interchange algorithm, and the complete deletion of gaps was implemented by MEGA 5 software. The percentage of replicate trees in which the associated taxa clustered together in the bootstrap test (500 replicates) are shown next to the branches. *S. pyogenes* was used as outgroup.
