# Supplementary material for: New insights on the biology of swine respiratory tract mycoplasmas from a comparative genome analysis
Source: BMC Genomics. 2013 Mar 14;14:175. doi: 10.1186/1471-2164-14-175 (PMC3610235; doi:10.1186/1471-2164-14-175)
Supplement: Additional file 19 — Evolutionary history of DNA methylases from mycoplasmas obtained through a phylogenetic analysis. The Neighbor-Joining method, using p-distance to compute the evolutionary distances and complete deletion of gaps was implemented by MEGA 5 software. The percentage of replicate trees in which the associated taxa clustered together in the bootstrap test (1,000 replicates) is shown next to the branches. [file 1471-2164-14-175-S19.pdf]

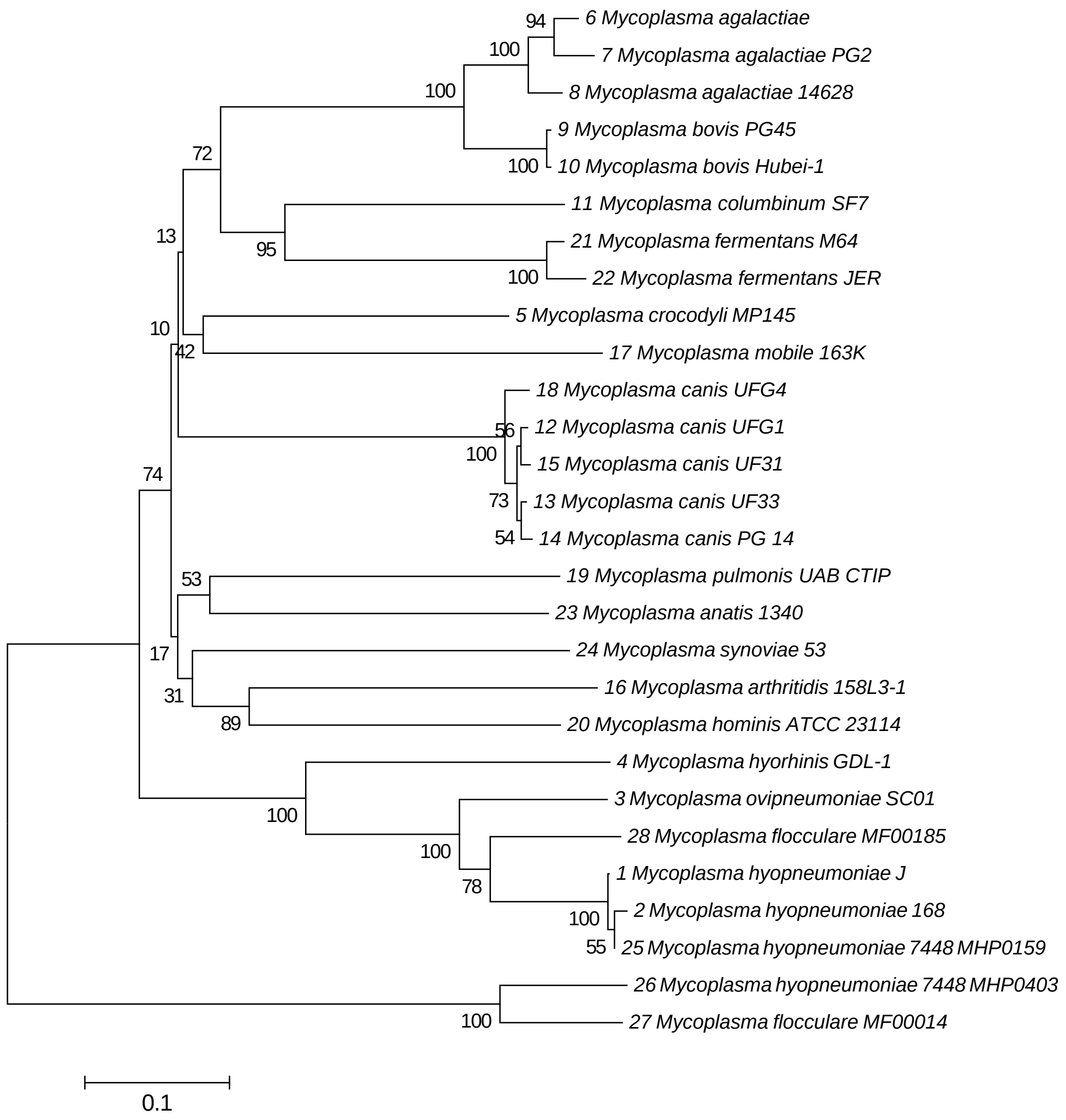

Additional file 19. **Evolutionary history of DNA methylases from mycoplasmas obtained through a phylogenetic analysis.** The Neighbor-Joining method, using p-distance to compute the evolutionary distances and pairwise deletion of gaps was implemented by MEGA 5 software. The percentage of replicate trees in which the associated taxa clustered together in the bootstrap test (1,000 replicates) is shown next to the branches.
