# Supplementary material for: New insights on the biology of swine respiratory tract mycoplasmas from a comparative genome analysis
Source: BMC Genomics. 2013 Mar 14;14:175. doi: 10.1186/1471-2164-14-175 (PMC3610235; doi:10.1186/1471-2164-14-175)
Supplement: Additional file 26 — Evolutionary history of glucose-6-phosphate-isomerase proteins from mycoplasmas obtained through a phylogenetic analysis. The Neighbor-Joining method was the same description of the Additional file 19. [file 1471-2164-14-175-S26.pdf]

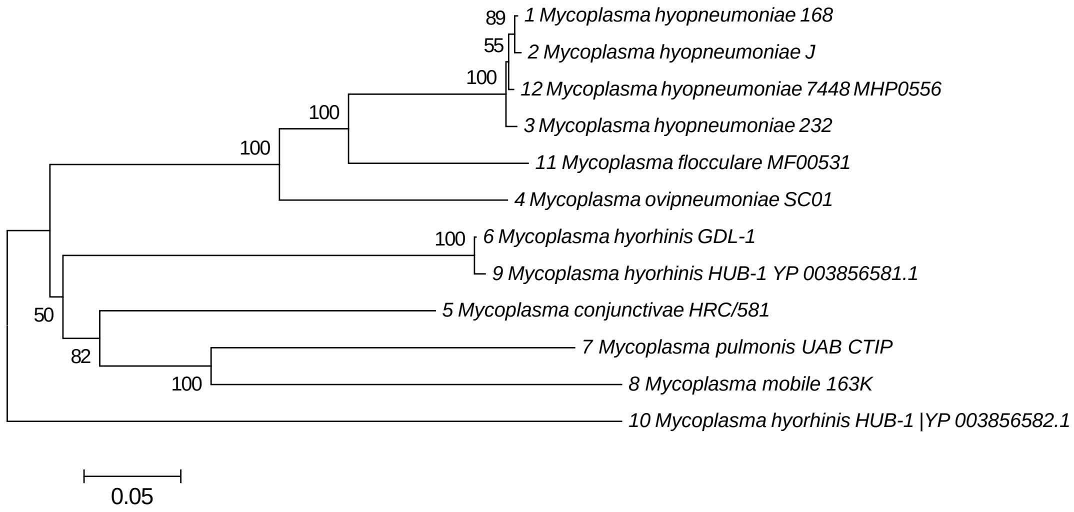

Additional file 26. Evolutionary history of glucose-6-phosphate-isomerase proteins from mycoplasmas obtained through a phylogenetic analysis. The Neighbor-Joining method was the same description of the Additional file 19.
