# Supplementary material for: New insights on the biology of swine respiratory tract mycoplasmas from a comparative genome analysis
Source: BMC Genomics. 2013 Mar 14;14:175. doi: 10.1186/1471-2164-14-175 (PMC3610235; doi:10.1186/1471-2164-14-175)
Supplement: Additional file 34 — Bacterial strains used in the phylogenomic analyses. [file 1471-2164-14-175-S34.pdf]

Additional file 34. Bacterial strains used in the phylogenomic analyses.

Strain	GenBank Accession no.	Genome size (Mb)	GC%	Protein
M. agalactiae	NC_013948.1	1.01	29.6	813
M. agalactiae PG2	NC_009497.1	0.88	29.7	742
M. arthritidis 158L3-1	NC_011025.1	0.82	30.7	631
M. bovis PG45	NC_014760.1	1.00	29.3	765
M. bovis Hubei-1	NC_015725.1	0.95	29.3	801
M. capricolum subsp. *capricolum* ATCC 27343	NC_007633.1	1.01	23.8	812
M. conjunctivae HRC/581	NC_012806.1	0.85	28.6	692
M. crocodyli MP145	NC_014014.1	0.93	27	689
M. fermentans M64	NC_014921.1	1.12	26.9	1,050
M. fermentans JER	NC_014552.1	0.98	26.9	797
M. flocculare		0.76	28.9	585
M. gallisepticum str. R(low)	NC_004829.2	1.01	31.5	763
M. genitalium G37	NC_000908.2	0.58	31.7	475
M. haemofelis str. Langford 1	NC_014970.1	1.15	38.9	1,545
M. hominis ATCC 23114	NC_013511.1	0.67	27.1	523
M. hyopneumoniae 232	NC_006360.1	0.89	28.6	691
M. hyopneumoniae 7422		0.89	28.4	692
M. hyopneumoniae 7448	NC_007332.1	0.92	28.5	657
M. hyopneumoniae J	NC_007295.1	0.9	28.5	657
M. hyorhinis HUB-1	NC_014448.1	0.84	25.9	658

M. leachii PG50	NC_014751.1	1.01	23.8	905
M. mobile 163K	NC_006908.1	0.78	25	633
M. mycoides subsp. Capri LC str. 95010	NC_015431.1	1.16	23.8	1,017
M. mycoides subsp. *mycoides* SC str. PG1	NC_005364.2	1.21	24	1,017
M. penetrans HF-2	NC_004432.1	1.36	25.7	1,037
M. pneumoniae M129	NC_000912.1	0.82	40	689
M. pulmonis UAB CTIP	NC_002771.1	0.96	26.6	782
M. putrefaciens KS1	NC_015946.1	0.83	26.9	650
M. suis KI3806	NC_015153.1	0.71	31.1	794
M. suis str. Illinois	NC_015155.1	0.74	31.1	845
M. synoviae 53	NC_007294.1	0.8	28.5	659
Streptococcus pyogenes M1 GAS	NC_002737	1.85	38.5	1,696
